# ERK1-mediated GLYCTK2 phosphorylation promotes fructolysis to sustain glioblastoma survival under glucose deprivation

**DOI:** 10.1038/s41420-025-02544-3

**Published:** 2025-06-04

**Authors:** Yingping Li, Fenna Zhang, Fumin Hu, Rui Tong, Yueqi Wen, Guokai Fu, Xueli Bian

**Affiliations:** 1 Department of Clinical Nutrition, The Second Affiliated Hospital, Jiangxi Medical College, Nanchang University, Nanchang, China; 2 The First Affiliated Hospital of Xi’an Medical University, Xi’an, China; 3 The MOE Basic Research and Innovation Center for the Targeted Therapeutics of Solid Tumors, School of Basic Medical Sciences, Jiangxi Medical College, Nanchang University, Nanchang, China

**Keywords:** Cancer metabolism, Phosphorylation

## Abstract

Metabolic plasticity sustains glioblastoma (GBM) survival under nutrient stress, yet how fructolytic adaptation compensates for glucose deprivation remains unclear. Here, we identify glycerate kinase 2 (GLYCTK2) as a glucose-sensing metabolic checkpoint that maintains GBM cell viability through ERK1-mediated phosphorylation. Mechanistically, glucose deprivation-activated ERK1 phosphorylates GLYCTK2 at serine 220 directly, which prevents STUB1 (ubiquitin E3 ligase) binding, thereby abrogating the ubiquitination and degradation of GLYCTK2. Importantly, Functional studies demonstrated that fructose supplementation rescues glucose deprivation-induced death in wild-type GBM cells, but fails to protect GLYCTK2-depleted cells, establishing GLYCTK2 as the gatekeeper of fructolytic salvage pathways. These findings demonstrate an important mechanism by which GBM cells rewire glucose metabolism to fructose metabolism via phosphorylating and stabilizing GLYCTK2 to maintain GBM cell survival under glucose deprivation condition, underscoring the potential to target GLYCTK2 for the treatment of patients with GBM.

## Introduction

Glioblastoma multiforme (GBM), the most prevalent and lethal primary brain malignancy, represents a therapeutic challenge due to its aggressive invasiveness, molecular heterogeneity, and near-universal recurrence. Recent epidemiological data from China highlight the disproportionate burden in pediatric populations, where brain tumors rank as the second most common malignancy among children aged 0–14 years [1]. Despite multimodal interventions including maximal safe resection, radiotherapy, and alkylating chemotherapy (e.g., temozolomide), clinical outcomes remain dismal with a median survival of 15 months and 5-year survival rates below 6% [2–4]. The therapeutic recalcitrance of GBM stems from its dual capacity for apoptotic evasion and adaptive metabolic reprogramming, mechanisms that remain incompletely understood. While immunotherapy and targeted therapies have shown preclinical promise, their clinical translation has been hindered by the absence of actionable biomarkers and a systems-level understanding of GBM’s molecular drivers. Crucially, the dynamic interplay between oncogenic signaling networks and cell death pathways, particularly those governing treatment resistance, remains underexplored.

Glucose serves as the primary substrate for cellular energy production and macromolecular biosynthesis in living organisms [5, 6], and dysregulation of its uptake or metabolism is implicated in the pathogenesis of diverse diseases, including cancer [7, 8]. A hallmark of cancer cell metabolism was first identified in 1924 by Otto Warburg, who observed that tumor cells preferentially undergo glycolysis for glucose utilization even in oxygen-rich conditions, a phenomenon termed the “Warburg effect” [9, 10]. This metabolic shift enables highly proliferative cancer cells to sustain their biosynthetic demands through increased extracellular glucose uptake. Paradoxically, despite this reliance on glycolysis, the tumor microenvironment (TME) of solid malignancies, including GBM, is frequently characterized by glucose scarcity [11–13], raising a fundamental question: How do cancer cells adapt to survive and proliferate under such nutrient-deprived conditions? Emerging evidence highlights metabolic reprogramming as a critical mechanism for cancer cell resilience under energy stress [14–17]. However, the precise molecular and metabolic adaptations employed by GBM cells to circumvent glucose deprivation remain poorly defined.

Glycerate kinase (GLYCTK) is a metabolic enzyme commonly expressed in prokaryotes and eukaryotes. There are two isoforms of GLYCTK in humans due to different alternative splicing of exons: GLYCTK1 and GLYCTK2. GLYCTK1 gene consists of a total of four exons (exons 2–5) and encodes 523 amino acids. Compared with GLYCTK1, GLYCTK2 terminates prematurely at the front of exon 5 due to codon shift caused by the lack of exon 4 and encodes 234 amino acids [18]. GLYCTK catalyzes the formation of 3-phosphate glycerol from glycerol in plants, fungi, and non autotrophic bacteria, while catalyzing the formation of 2-phosphate glycerol from glycerol in animals and methylotrophic bacteria [19–22]. It was found that GLYCTK is widely expressed in various tissues of the human body and GLYCTK2 is the dominant expressed isoform via PCR identification in 17 human tissues. GLYCTK is mainly expressed in the hippocampal neurons of the cerebral cortex, corpus callosum, and brain tissue in mice. The inactivation of GLYCTK can lead to a metabolic disease called D-Glyceric Acidemia/Aciduria in clinical practice [23–27], which is characterized by delayed mental development, intellectual disability and language disorders [28]. A large amount of glycerol acid accumulation was detected in their blood and urine in patients with glycerol acidosis after administration of serine and fructose [29], implying that glycerol acid accumulation is the result of a shared reaction between serine and fructose metabolism. GLYCTK catalyzes the formation of 2-phosphoglycerol from glycerol which is a intermediate of serine and fructose catabolism pathway which has been reported to play vital role in cancer cell progression [30–33]. However, whether GLYCTK2 is involved in GBM survival through fructose catabolic pathway under glucose deprivation is unknown.

In this study, we reveal that glucose deprivation induces ERK1-mediated phosphorylation of GLYCTK2 at S220, which antagonizes STUB1-dependent ubiquitination and subsequent proteasomal degradation. Stabilized GLYCTK2 drives adaptive fructolytic flux to sustain GBM survival under metabolic stress, thereby identifying a targetable vulnerability in GBM’s metabolic plasticity through its reliance on glycerate kinase-mediated compensatory pathways.

## Results

### GLYCKT2 is overexpressed in GBM and promotes GBM tumorigenesis

**Fig. 1:**
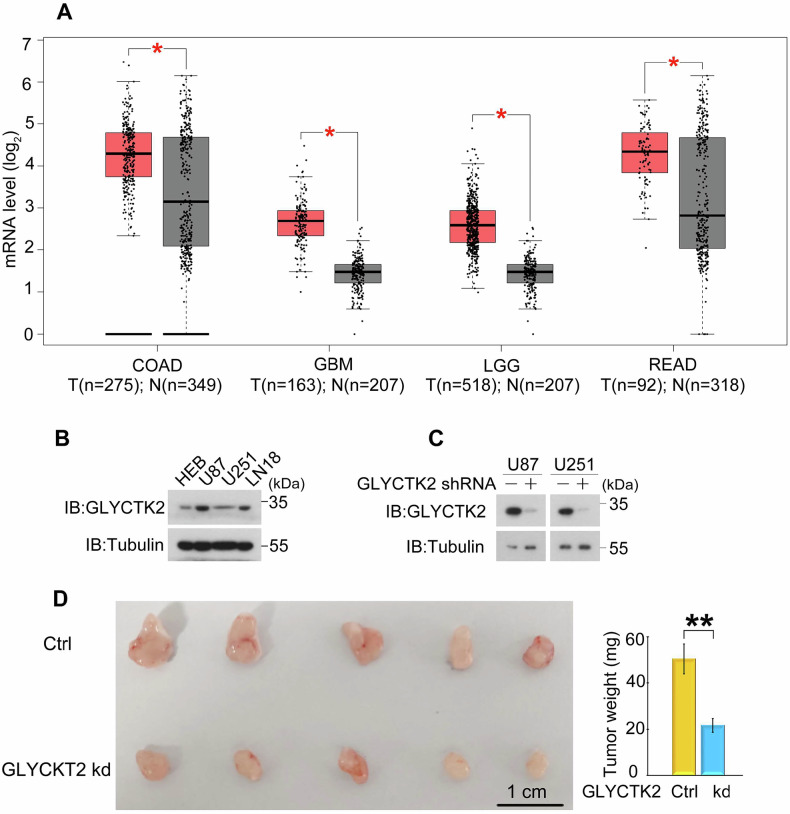
GLYCKT2 promotes GBM tumorigenesis. **A** Quantitative analysis of *GLYCTK* mRNA level in tumor (T) versus normal (N) tissues from the GEPIA cohort. Data are presented as boxplots, *p < 0.05. **B** Immunoblot analysis of GLYCTK2 protein expression in multiple glioblastoma cell lines. Tubulin served as a loading control. **C** Validation of GLYCTK2 knockdown efficiency in U87 and U251 glioblastoma cells transduced with lentiviral shRNA constructs. Tubulin was used for normalization. **D** Tumorigenic capacity of GLYCTK2-depleted U87 cells in vivo. Control or GLYCTK2-knockdown cells were implanted subcutaneously into athymic nude mice (*n* = 5/group). Tumors were harvested at day 16 post-implantation, with representative images (left) and tumor weights (right) shown. **p < 0.01 (Student’s *t* test).

To systematically investigate the oncogenic potential of GLYCTK2 in cancer progression, we initially analyzed its transcriptional profile across pan-cancer datasets using the Gene Expression Profiling Interactive Analysis (GEPIA) platform (http://gepia.cancer-pku.cn/). Comparative analysis revealed significant upregulation of *GLYCTK2* mRNA in colon adenocarcinoma (COAD), glioblastoma multiforme (GBM), brain lower-grade glioma (LGG), and rectum adenocarcinoma (READ) compared with matched normal tissues (Fig. 1A and Supplementary Fig. 1). The co-occurrence of LGG and GBM as brain malignancies underscores the critical regulatory role of GLYCTK2 in driving malignant progression within brain tumors. Given the clinical significance of GBM as the most prevalent and aggressive primary brain malignancy, we focused subsequent investigations on its role in glioblastoma progression. Experimental validation through immunoblotting confirmed constitutive overexpression of GLYCTK2 protein in multiple GBM cell lines (U87, U251, LN18) relative to normal human astrocytes (HEB) (Fig. 1B). To establish functional causality, we generated stable GLYCTK2-knockdown models using lentiviral-mediated shRNA in GBM cells (Fig. 1C) and orthotopic xenograft models demonstrated that GLYCTK2 depletion significantly attenuated tumor growth in vivo (Fig. 1D). These data demonstrate that GLYCTK2 is overexpressed in GBM and promotes GBM progression.

### GLYCTK2 depletion impairs glioblastoma cell survival under glucose deprivation

**Fig. 2:**
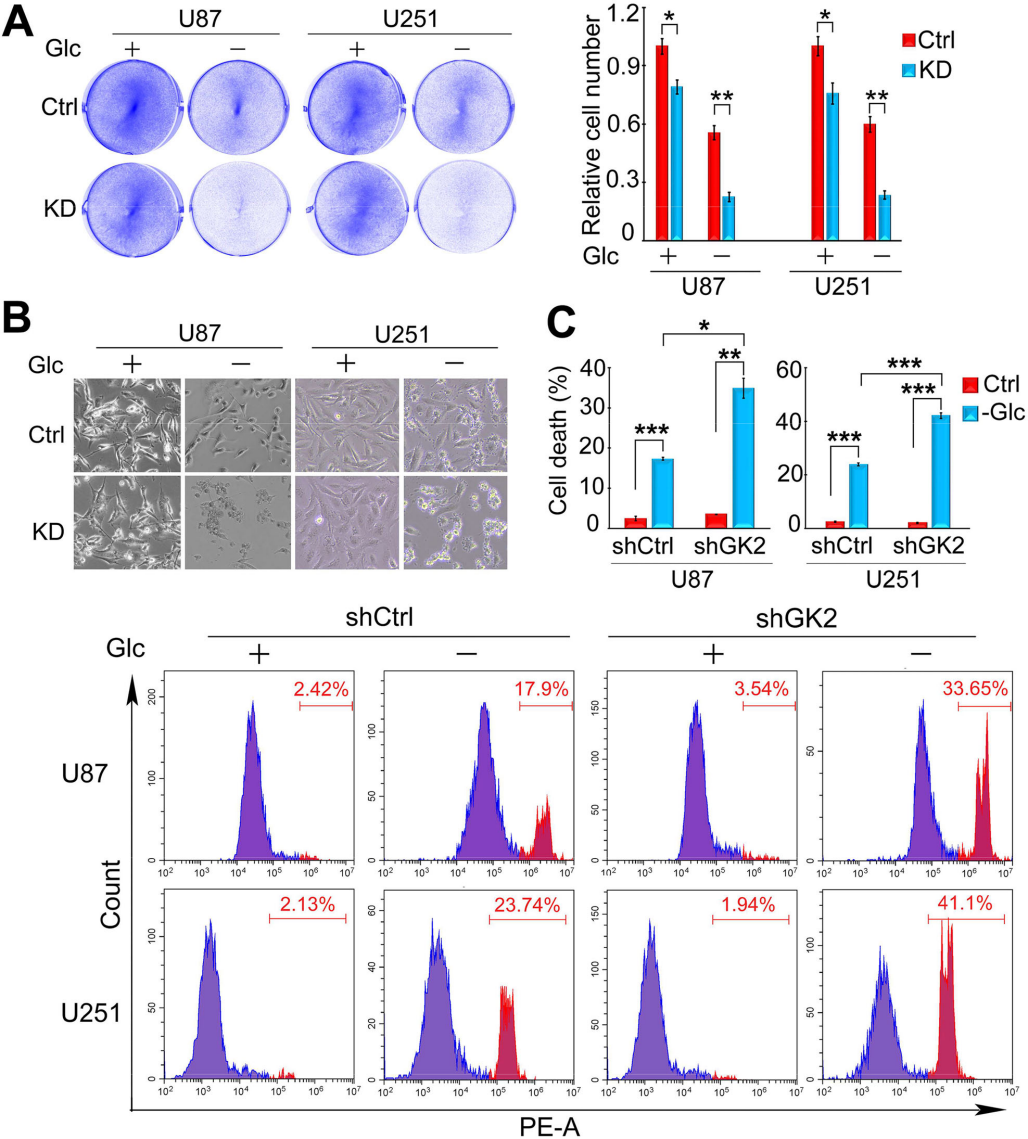
GLYCTK2 depletion impairs glioblastoma cell survival under glucose deprivation. Viability analysis of control (Ctrl) and *GLYCTK2*-knockdown (KD) glioblastoma cells under glucose-restricted conditions. Cells were cultured in glucose-containing (+Glc) or glucose-free (−Glc) medium for 16 h, followed by crystal violet staining (**A**) or phase-contrast microscopy imaging (**B**). The crystal violet-stained cells were eluted with 10% glacial acetic acid and measured at 595 nm using a microplate reader (**A**, right). Data represent the means ± SEM of 3 independent experiments. * *p* < 0.05,** *p* < 0.01 (Student’s *t* test). **C** Quantification of glucose deprivation-induced cell death by flow cytometry. Control (shCtrl) and *GLYCTK2*-knockdown (shGLYCTK2) cells were treated with ±Glc for 12 h, stained with propidium iodide (PI), and analyzed in the phycoerythrin (PE) channel. Top: Representative flow cytometry density plots gated on PI-positive populations. Bottom: Statistical analysis of cell death rates across three independent experiments (mean ± SEM). *p < 0.05, **p < 0.01, ***p < 0.001 (one-way ANOVA).

While macromolecule biosynthesis and energy metabolism constitute fundamental requirements for cellular proliferation, tumor cells exhibit remarkable plasticity through metabolic reprogramming under nutrient-deficient conditions. However, the precise molecular regulators governing this adaptive process remain poorly characterized. To investigate the mechanistic basis of GLYCTK2 in GBM survival under metabolic stress, we subjected control and GLYCTK2-depleted cells to 16-h glucose deprivation. Colony formation revealed that GLYCTK2 knockdown suppresses GBM cell proliferation under glucose-supplemented conditions, with a more pronounced inhibitory effect observed in glucose-deprived conditions (Fig. 2A). The modest reduction in colony formation under standard glucose conditions may reflect a partial dependency of baseline proliferation on GLYCTK2-mediated metabolic activity. It could suggest that GLYCTK2 supports cellular fitness even in nutrient-replete environments. Intriguingly, phase-contrast microscopy demonstrated a marked accumulation of non-adherent, rounded cells in GLYCTK2-deficient populations during glucose deprivation (Fig. 2B), phenotypically indicative of cell death progression. To quantitatively assess the cell death rate, propidium iodide (PI) staining coupled with flow cytometric analysis confirmed that GLYCTK2 silencing exacerbated glucose deprivation-induced GBM cell death rate (Fig. 2C). These data establish GLYCTK2 as a critical mediator of metabolic adaptation, sustaining GBM proliferation and survival under glucose deprivation condition.

### GLYCTK2 modulates fructose-dependent survival in glucose-deprived GBM cells

**Fig. 3:**
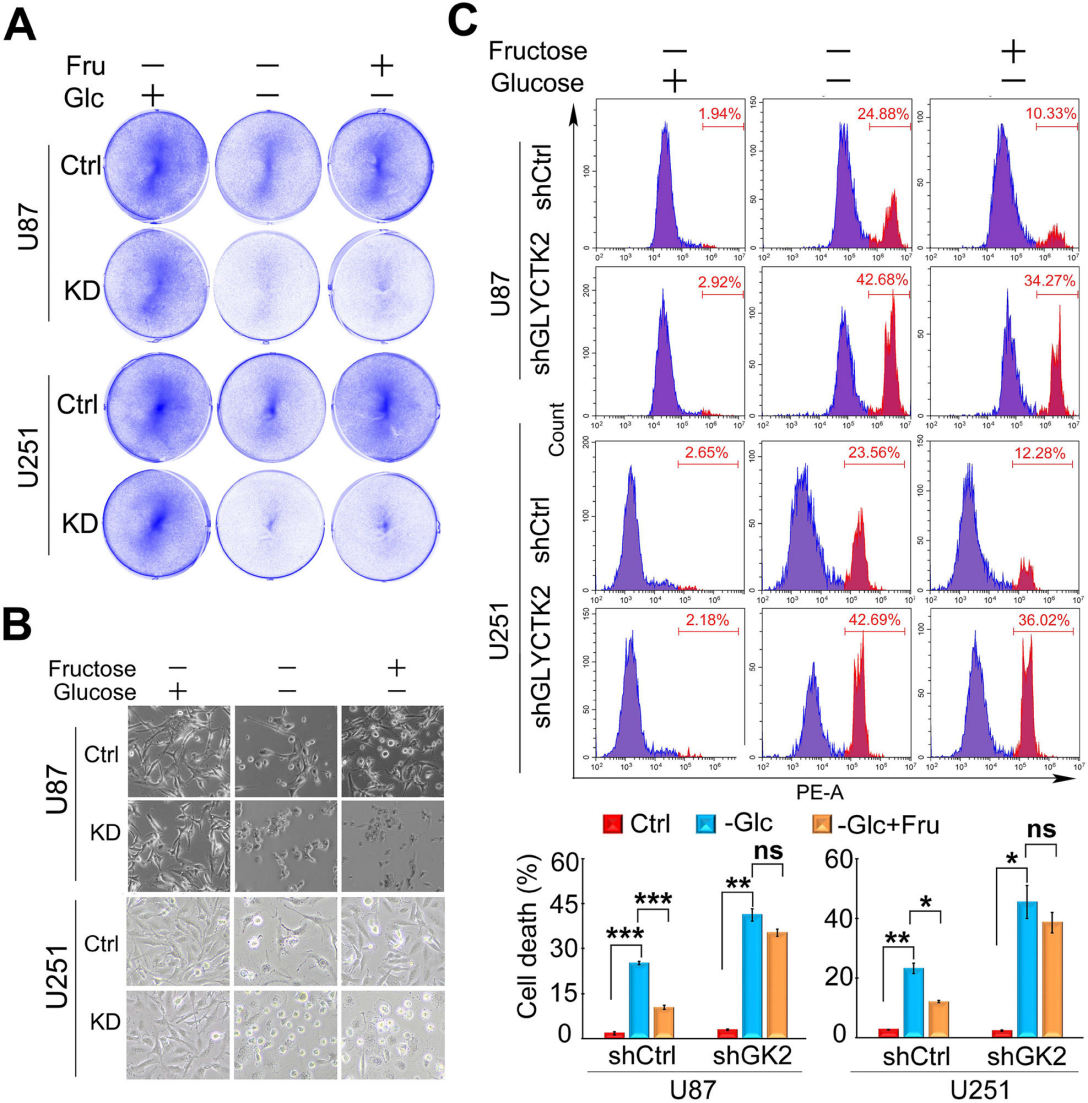
GLYCTK2 modulates fructose-dependent survival in glucose-deprived GBM cells. Viability of control (shCtrl) or *GLYCTK2*-knockdown (shGLYCTK2) GBM cells. GBM cells were cultured with or without glucose (Glc) in the presence or absence of 10 mM fructose (Fru) for 16 h followed by staining with crystal violet (**A**) or photographed (**B**). **C** Cell death analysis by propidium iodide (PI) staining. Control (shCtrl) or GLYCTK2 knockdown (shGLYCTK2) GBM cells were cultured with or without glucose in the presence or absence of 10 mM fructose for 12 h followed by staining with PI and analyzed with flow cytometry in the channel of PE. Representative flow cytometric images (top) and calculated cell death rate (bottom) are shown, GK2 GLYCTK2, Glc glucose, Fru fructose. Data represent the means ± SEM of 3 independent experiments. *p < 0.05, **p < 0.01, ***p < 0.001, ns: no significance (One-way ANOVA).

Our findings establish that GLYCTK2 is essential for maintaining GBM cell viability under glucose deprivation. As a metabolic enzyme, GLYCTK2 catalyzes the conversion of glycerate (a catabolic byproduct of serine/fructose metabolism) to 3-phosphoglycerate, thereby replenishing glycolytic intermediates. Given the emerging role of dietary fructose in promoting oncogenesis, we hypothesized that GBM cells may employ GLYCTK2-dependent fructose catabolism to circumvent glucose scarcity. To interrogate this metabolic adaptation mechanism, control and GLYCTK2-depleted GBM cells were maintained in glucose-free DMEM supplemented with or without 10 mM fructose. Colony formation assays demonstrated that fructose supplementation significantly rescued proliferation inhibition caused by glucose deprivation in control cells, but failed to restore growth in GLYCTK2-knockdown populations (Fig. 3A). Consistent with the proliferation data, PI staining revealed that fructose attenuated glucose deprivation-induced cell death in control GBM cells, while showing no protective effect in GLYCTK2-deficient cells (Fig. 3B, C). SLC2A5, the primary fructose transporter, and ketohexokinase (KHK), the rate-limiting enzyme in fructose catabolism, are key molecular players in fructose metabolism. To determine whether GLYCTK2 regulates fructose catabolism by modulating the expression of these proteins, we performed Western blot analysis. Results revealed that glucose deprivation induced a marginal upregulation of SLC2A5 but had no detectable effect on KHK. Importantly, both SLC2A5 and KHK protein levels remained comparable between shControl (shCtr) and GLYCTK2-knockdown (shGLYCTK2) cells under these conditions (Supplementary Fig. 2A). However, GLYCTK2 knockdown impaired fructose uptake (Supplementary Fig. 2B). Notably, while GLYCTK2 depletion did not affect SLC2A5 expression, we propose that the observed suppression of fructose utilization stems from disrupted fructose catabolism. As a key enzyme in fructose degradation, GLYCTK2 knockdown likely inhibits fructose breakdown, consequently reducing both its metabolic utilization and compensatory uptake through feedback regulation. Taken together, these results delineate a critical role for GLYCTK2 in mediating metabolic plasticity, where GLYCTK2-mediated fructose catabolism serves as a compensatory metabolic axis to sustain tumor cell survival under glucose deprivation.

### Glucose deprivation stabilizes GLYCTK2

**Fig. 4:**
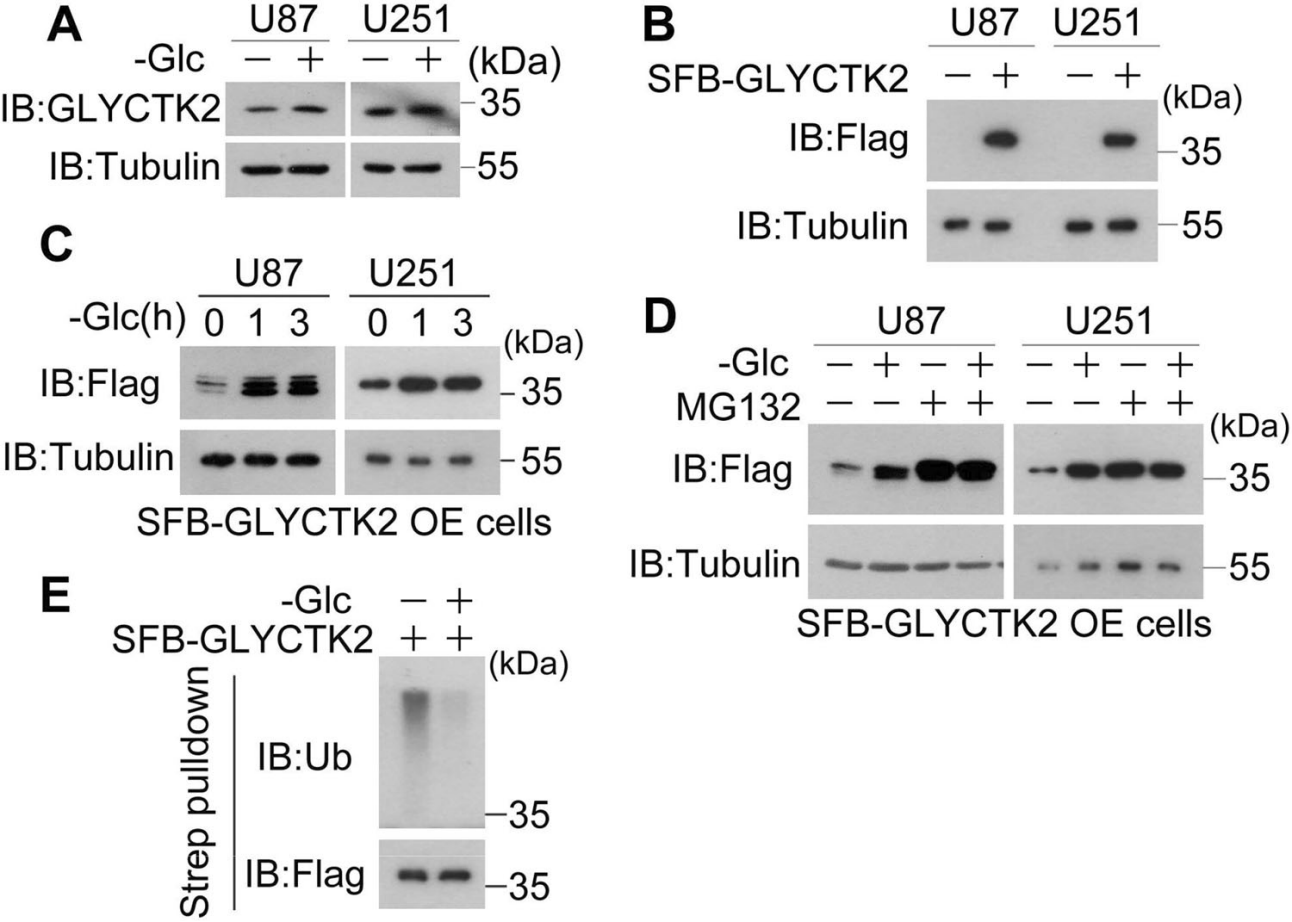
Glucose deprivation stabilizes GLYCTK2. **A** Immunoblot analysis of U87 and U251 glioblastoma cells following 3-hour glucose deprivation. Proteins were detected using antibodies against indicated targets. **B** Validation of stable SFB-GLYCTK2 expression in U87 and U251 cell lines by immunoblotting with specified antibodies. **C** Immunoblot analysis of SFB-GLYCTK2 overexpression dynamics under glucose deprivation for different periods in U87 and U251 cells (Glc glucose, OE overexpression). **D** SFB-GLYCTK2 overexpressed U87 and U251 cells stimulated with or without glucose deprivation in the presence or absence of 10 μM MG132 for 3 h were harvested for immunoblot analysis with indicated antibodies (Glc glucose, OE overexpression). **E** Streptavidin affinity precipitation followed by immunoblot analysis of SFB-GLYCTK2 ubiquitination in U87 cells treated with 10 μM MG132 under glucose deprivation for 3 h. **A**–**D** Tubulin served as a loading control.

Next we evaluate the dynamic regulation of GLYCTK2 under glucose deprivation, GBM cells were cultured with or without glucose for 3 h, western blot (WB) analyses showed that glucose deprivation increases endogenous GLYCTK2 protein level in U87 and U251 cells (Fig. 4A). To dissect the regulatory mechanism governing this phenomenon, we established stable cell lines expressing SFB-tagged GLYCTK2 in U87 and U251 cells (Fig. 4B) and found that glucose deprivation also increased exogenous GLYCTK2 protein level (Fig. 4C). Because the promoter sequences of endogenous and exogenous GLYCTK2 are different, these observation implies that the accumulated protein level under glucose deprivation may be regulated at post-translational level. Consistently, MG132, the ubiquitin-proteasome degradation pathway inhibitor, can eradicate glucose deprivation-induced GLYCTK2 accumulation in U87 and U251 cells (Fig. 4D). Importantly, ubiquitination assay revealed that glucose deprivation significantly inhibits GLYCTK2 poly-ubiquitination level (Fig. 4E). Collectively, these data establish that glucose deprivation enhances GLYCTK2 stability through suppression of ubiquitin-mediated proteolysis, revealing a novel adaptive mechanism in tumor metabolic reprogramming.

### ERK signaling pathway regulates GLYCTK2 stability under glucose deprivation

**Fig. 5:**
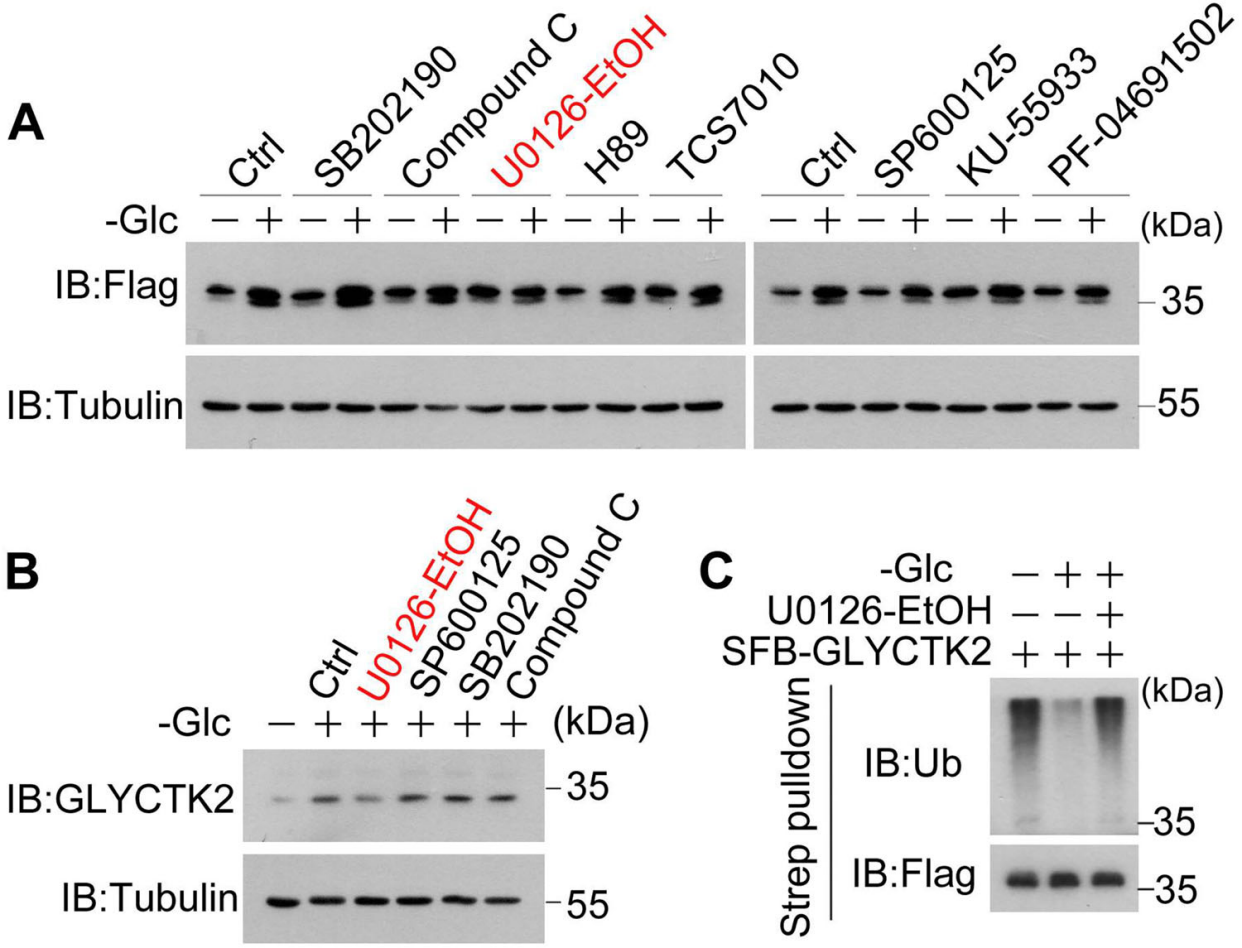
ERK signaling pathway regulates GLYCTK2 stability under glucose deprivation. **A** U87 cells overexpressing SFB-tagged GLYCTK2 stimulated with or without glucose deprivation in the presence or absence of 20 μM SB202190 (p38 inhibitor), 10 μM Compound C (AMPK inhibitor), 20 μM U0126-EtOH (ERK inhibitor), 20 μM H89 (PKA inhibitor), 2.5 μM TSC7010 (Aurora A Inhibitor I), 25 μM SP600125 (JNK inhibitor), 20 μM KU-55933 (ATM inhibitor) or 1 μM PF-04691502 (PI3K/ mTOR inhibitor) for 3 h were harvested for immunoblot analysis with indicated antibodies, Tubulin as a loading control. **B** U87 cells stimulated with or without glucose deprivation in the presence or absence of 20 μM U0126-EtOH (ERK inhibitor), 25 μM SP600125 (JNK inhibitor), 20 μM SB202190 (p38 inhibitor) or 10 μM Compound C (AMPK inhibitor) for 3 h were harvested for immunoblot analysis with indicated antibodies. Tubulin served as a loading control. **C** U87 cells overexpressing SFB-tagged GLYCTK2 stimulated with or without glucose deprivation in the presence or absence of 20 μM U0126-EtOH for 3 h were harvested and immunoprecipitated with streptavidin (Strep) agarose for immunoblot analysis with indicated antibodies.

While glucose serves as the primary metabolic substrate for cellular processes, emerging evidence indicates that glucose deprivation triggers adaptive signaling through kinase cascades [34–36]. To systematically identify regulatory kinases governing GLYCTK2 protein stability during glucose deprivation, we employed a pharmacological screening approach using selective kinase inhibitors in U87 cells cultured in glucose-free DMEM. Immunoblotting analysis revealed that U0126-EtOH (MEK/ERK pathway inhibitor) specifically abrogated glucose deprivation-induced elevation of FLAG-tagged GLYCTK2 protein level (Fig. 5A). In line with this finding, U0126-EtOH also inhibits the upregulation of endogenous GLYCTK2 protein level induced by glucose deprivation (Fig. 5B). Because glucose deprivation inhibits GLYCTK2 ubiquitination and degradation in GBM cells (Fig. 4D, E), ubiquitination assay showed that pretreatment with ERK inhibitor U0126-EtOH attenuates glucose deprivation-induced downregulation of poly-ubiquitination level of GLYCTK2 (Fig. 5C). These data suggest that ERK activation stimulated by glucose deprivation is responsible for GLYCTK2 stabilization in GBM cells.

### E3 ubiquitin ligase STUB1 stabilizes GLYCTK2

**Fig. 6:**
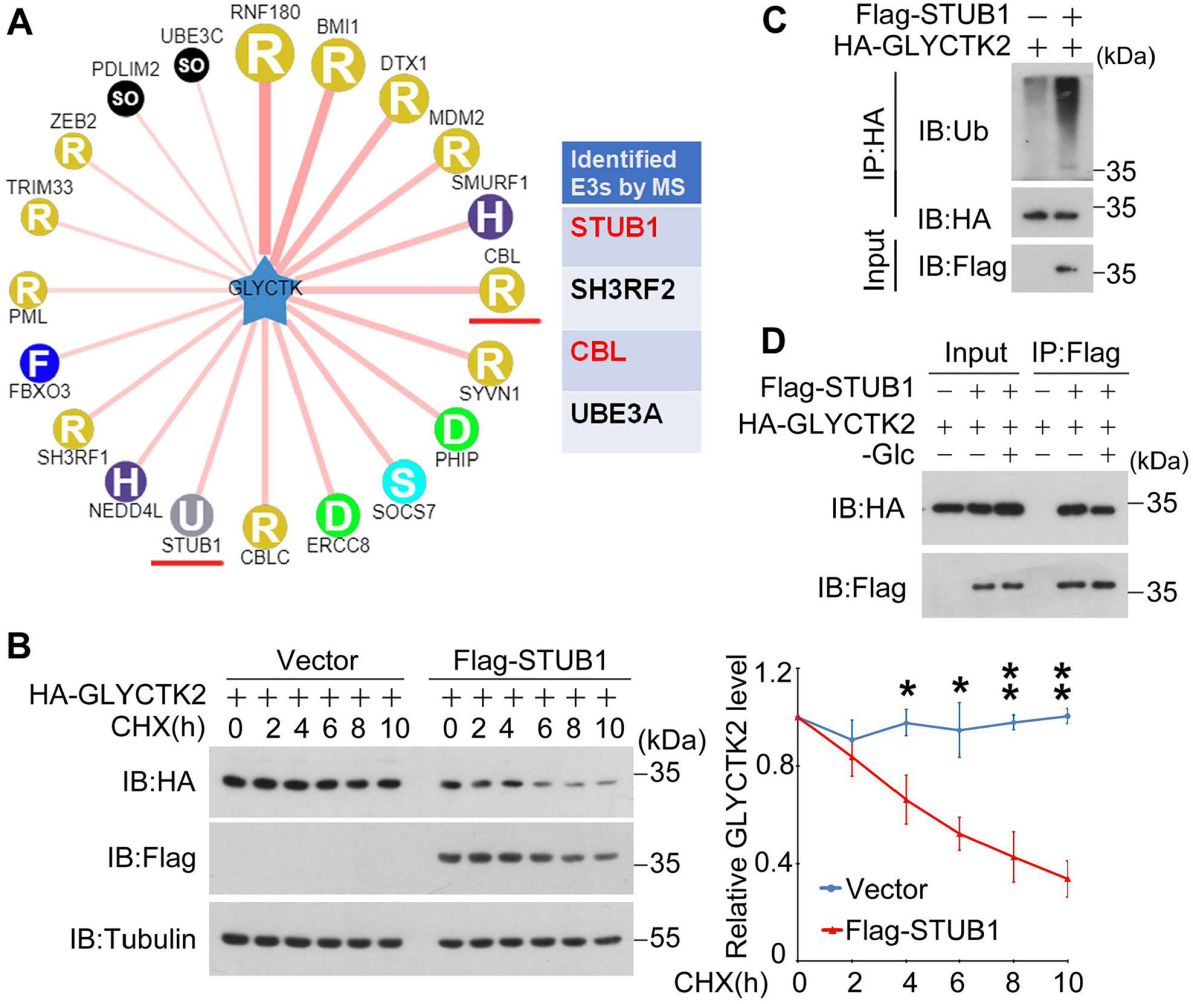
E3 ubiquitin ligase STUB1 stabilizes GLYCTK2. **A** Left: Computational prediction of potential ubiquitin E3 ligases interacting with GLYCTK using Ubibrowser (http://ubibrowser.bio-it.cn/ubibrowser/). Right: Mass spectrometry-based identification of GLYCTK2-associated E3 ligases in U87 glioblastoma cells. **B** Protein stability analysis of GLYCTK2 in U87 cells. Left: Western blot of cells transfected with specified constructs for 24 h, followed by cycloheximide (CHX, 100 μg/mL) treatment for indicated durations. Right: Quantification of GLYCTK2 protein levels normalized to initial levels (0 h). Data represent mean ± SEM of three independent experiments. * *p* < 0.05, ** *p* < 0.01 (Student’s *t* test). **C** U87 cells transfected with indicated plasmids for 24 h were treated with 10 μM MG132 (proteasome inhibitor) for an additional 6 h. Protein complexes were immunoprecipitated (IP) using anti-HA antibodies and analyzed by Western blotting with indicated antibodies. **D** U87 cells transfected with specified plasmids for 24 h were subjected to glucose deprivation or control conditions for 3 h. Protein complexes were immunoprecipitated with anti-FLAG antibodies and analyzed by Western blotting with indicated antibodies.

Given above results indicating that GLYCTK2 is stabilized under glucose deprivation, this finding raised the question of how glucose deprivation participates in GLYCTK2 stability. To elucidate this question, we immunoprecipitated GLYCTK2 to identify its interaction partners through mass spectrometry, hundreds of proteins were identified and the ubiquitin E3 ligase SUTB1 and CBL caused our attention because these two proteins are also predicted as the ubiquitin E3 ligase candidates of GLYCTK2 with UbiBrowser (http://ubibrowser.bio-it.cn/ubibrowser/) (Fig. 6A). To identify which ubiquitin E3 ligase is responsible for GLYCTK2 stability, we overexpressed these two proteins separately and found that overexpression of STUB1 (Fig. 6B) but not CBLB (Supplementary Fig. 3), significantly shorten the half-life of GLYCTK2. Of note, overexpression of STUB1 significantly increases the poly-ubiquitination level of GLYCTK2 (Fig. 6C), implying that STUB1 may be the ubiquitin E3 ligase of GLYCTK2. Importantly, STUB1 interacts with GLYCTK2 and its association was weakened under glucose deprivation condition (Fig. 6D). These data suggest that glucose deprivation may stabilize GLYCTK2 via inhibiting its interaction with ubiquitin E3 ligase STUB1.

### ERK1 phosphorylates GLYCTK2 at S220 to promotes its stability

**Fig. 7:**
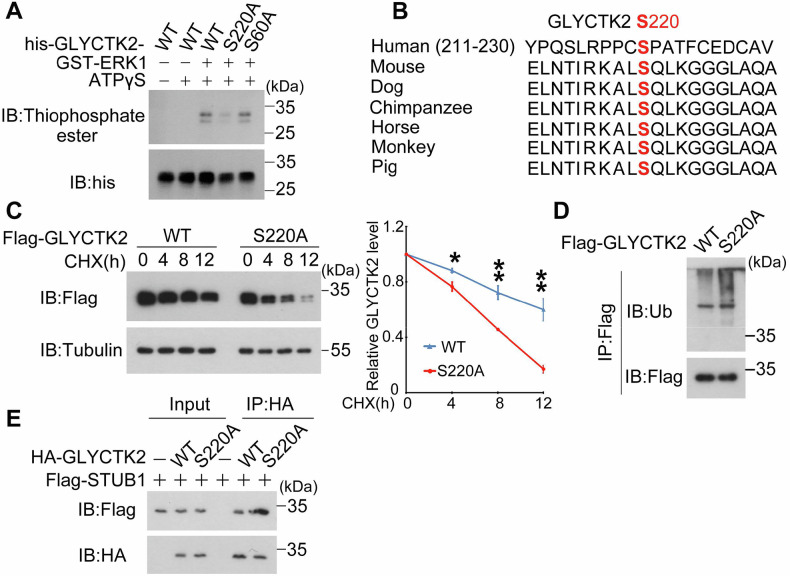
ERK1 phosphorylates GLYCTK2 to promotes its stability. **A** In vitro kinase assay of ERK1-mediated GLYCTK2 phosphorylation. Recombinant His-tagged wild-type (WT) GLYCTK2 or its mutant proteins purified from *E.coli* were incubated with or without activated GST-ERK1 and ATPγS in kinase buffer. Phosphorylation status was assessed by immunoblotting (IB) with indicated antibodies. **B** Alignment of protein sequences spanning GLYCTK2 S220 from different species. **C** S220 phosphorylation stabilizes GLYCTK2 protein. (Left) U87 cells transfected with Flag-tagged WT GLYCTK2 or S220A mutant were treated with cycloheximide (CHX, 100 μg/mL) for specified durations (0–12 h), followed by IB analysis. Tubulin served as a loading control. (Right) Quantification of GLYCTK2 protein half-life normalized to initial level (0 h; *p < 0.05, **p < 0.01; Student’s t-test; mean ± SEM, *n* = 3). **D** U87 cells expressing Flag-GLYCTK2 WT or S220A mutant were harvested for immunoprecipitation with anti-Flag antibodies followed by WB analysis with indicated antibodies. **E** U87 cells transfected with indicated plasmids for 30 h were harvested for immunoprecipitation with anti-HA antibodies followed by WB analysis with indicated antibodies.

The observed ERK-dependent stabilization of GLYCTK2 prompted investigation into the molecular crosstalk between kinase signaling and protein degradation. As a canonical serine/threonine kinase, ERK modulates substrate function through phosphorylation. Using Scansite 4.0 (https://scansite4.mit.edu/#scanProtein), we identified S220 of GLYCTK2 as a putative ERK1 phosphorylation site (Supplementary Fig. 4). To clarify whether ERK1 phosphorylates GLYCTK2 S220 directly, we conducted in vitro kinase assay with purified WT, S220A or S60A (as a control) GLYCTK2 in *Escherichia coli* (*E.coli*) and results showed that ERK1 can phosphorylate WT or S60A GLYCTK2 but not S220A mutant (Fig. 7A), suggesting that ERK1 may directly phosphorylate GLYCTK2 at S220. Protein sequence alignment showed that GLYCTK2 S220 is highly conserved in various species (Fig. 7B), implying that S220 may be a critical site for GLYCTK2 function. To determine whether S220 phosphorylation is responsible for the regulation of GLYCTK2 stability, CHX (cycloheximide) chase assay was used and results showed that compared to WT GLYCTK2, S220A mutant has a shorter half-life (Fig. 7C). Consistent with this finding, the poly-ubiquitination level of S220A GLCYTK2 is much higher than WT GLYCTK2 (Fig. 7D), implying that S220 phosphorylation may increase GLYCTK2 stability. Given our results indicating that E3 ubiquitin ligase STUB1 was responsive for GLYCTK2 degradation, finally, we want to gain insight into the mechanism of S220 phosphorylation on GLYCTK2 stability, co-immunoprecipitation assay was conducted and result showed that compared to WT GLYCTK2, the phosphorylation mutant GLYCTK2 S220A has a higher affinity with the ubiquitin E3 ligase STUB1 (Fig. 7E). These findings demonstrate that S220 phosphorylation by ERK1 is responsible for GLYCTK2 stability via inhibiting its interaction with STUB1 under glucose deprivation.

### ERK1-mediated S220 phosphorylation of GLYCTK2 promotes glioblastoma survival

**Fig. 8:**
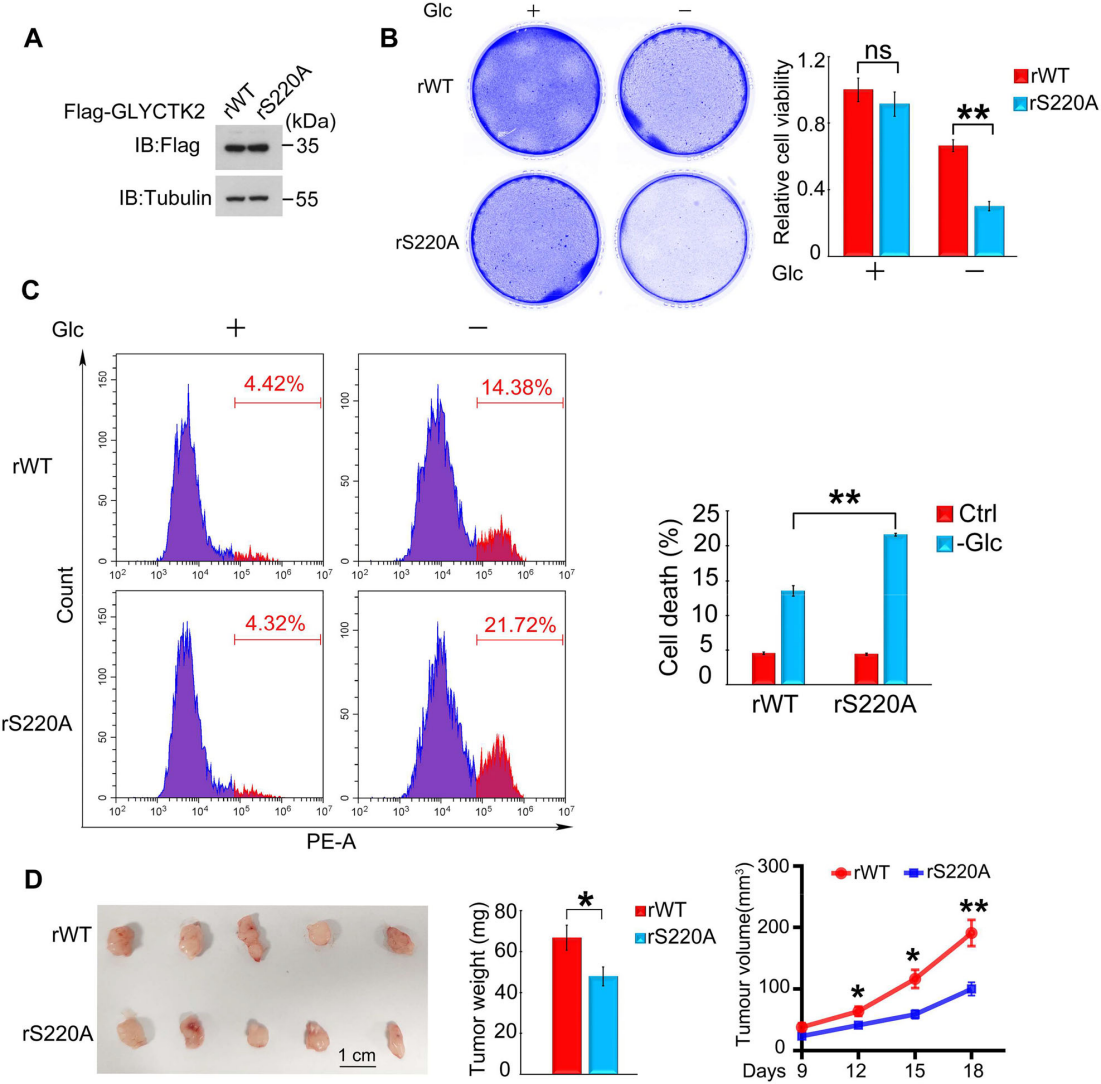
Phosphorylation of GLYCTK2 at S220 promotes glioblastoma survival. **A** Validation of GLYCTK2 rescue constructs in U87 cells. GLYCTK2-knockdown U87 cells were stably transfected with shRNA-resistant, Flag-tagged wild-type GLYCTK2 (rWT) or the S220A mutant (rS220A). Western blot (WB) analysis confirms the expression level of rescued GLYCTK2 variants. **B** Viability of rWT- or rS220A-overexpressing U87 cells was assessed under glucose-containing (+Glc) or glucose-free (−Glc) conditions for 16 h. Cells were subjected to crystal violet staining (left), followed by quantification of retained dye after elution with 10% glacial acetic acid (absorbance measured at 595 nm; right). Data are presented as mean ± SEM (*n* = 3 independent experiments). **p < 0.01 (Student’s t-test). **C** Flow cytometric quantification of cell death in rWT or rS220A U87 cells treated with ± Glc for 12 h. Cells were stained with propidium iodide (PI) and analyzed in the phycoerythrin (PE) channel. Left: Representative density plots showing PI-positive populations. Right: Quantitative analysis of cell death rates (mean ± SEM; *n* = 3). *p < 0.05, **p < 0.01, ***p < 0.001 (Student’s t-test). **D** GLYCTK2 S220A mutation attenuates tumor growth in vivo. GLYCTK2-knockdown U87 cells stably expressing rWT or rS220A were subcutaneously inoculated into nude mice (*n* = 5/group). Tumors were excised 18 days post-injection. Left: Representative tumor photographs. Middle: Tumor weight. Right: Tumor volume (mean ± SEM). *p < 0.05, **p < 0.01 (Student’s t-test).

To investigate whether ERK1-mediated phosphorylation of GLYCTK2 at serine 220 under glucose deprivation regulates glioblastoma cell survival, we established GLYCTK2-knockdown U87 cells stably overexpressing shRNA-resistant Flag-tagged wild-type GLYCTK2 (rWT) or the non-phosphorylatable S220A mutant (rS220A) (Fig. 8A). Following 16-hour glucose starvation, colony formation assays revealed a significant impairment in clonogenic capacity in rS220A-expressing cells compared to rWT controls (Fig. 8B). Concordantly, cell death assays demonstrated markedly increased mortality in rS220A-expressing cells under glucose deprivation relative to rWT-expressing counterparts (Fig. 8C). To further interrogate the role of S220 phosphorylation in glioblastoma progression, subcutaneous xenograft models were employed. Strikingly, tumors derived from rS220A-expressing U87 cells exhibited substantial reductions in both tumor weight and volume compared to rWT-derived tumors (Fig. 8D). Collectively, these findings establish that ERK1-mediated phosphorylation of GLYCTK2 at S220 promotes glioblastoma survival during glucose starvation.

## Discussion

Glioblastoma multiforme remains one of the most aggressive malignancies with limited therapeutic efficacy, primarily due to its metabolic adaptability and resistance to conventional treatments [37, 38]. The identification of critical metabolic vulnerabilities in GBM cells therefore represents an urgent priority for developing targeted therapies. While metabolic reprogramming is a hallmark of cancer, recent evidence suggests that tumor cells exhibit remarkable plasticity in utilizing alternative carbon sources under glucose-restricted conditions [33, 39, 40]. Notably, fructose, a hexose abundant in dietary sources, has emerged as a potential substrate for tumor progression through the fructolytic pathway. However, the molecular mechanisms underlying fructose-driven metabolic adaptation in GBM remain poorly characterized.

**Fig. 9:**
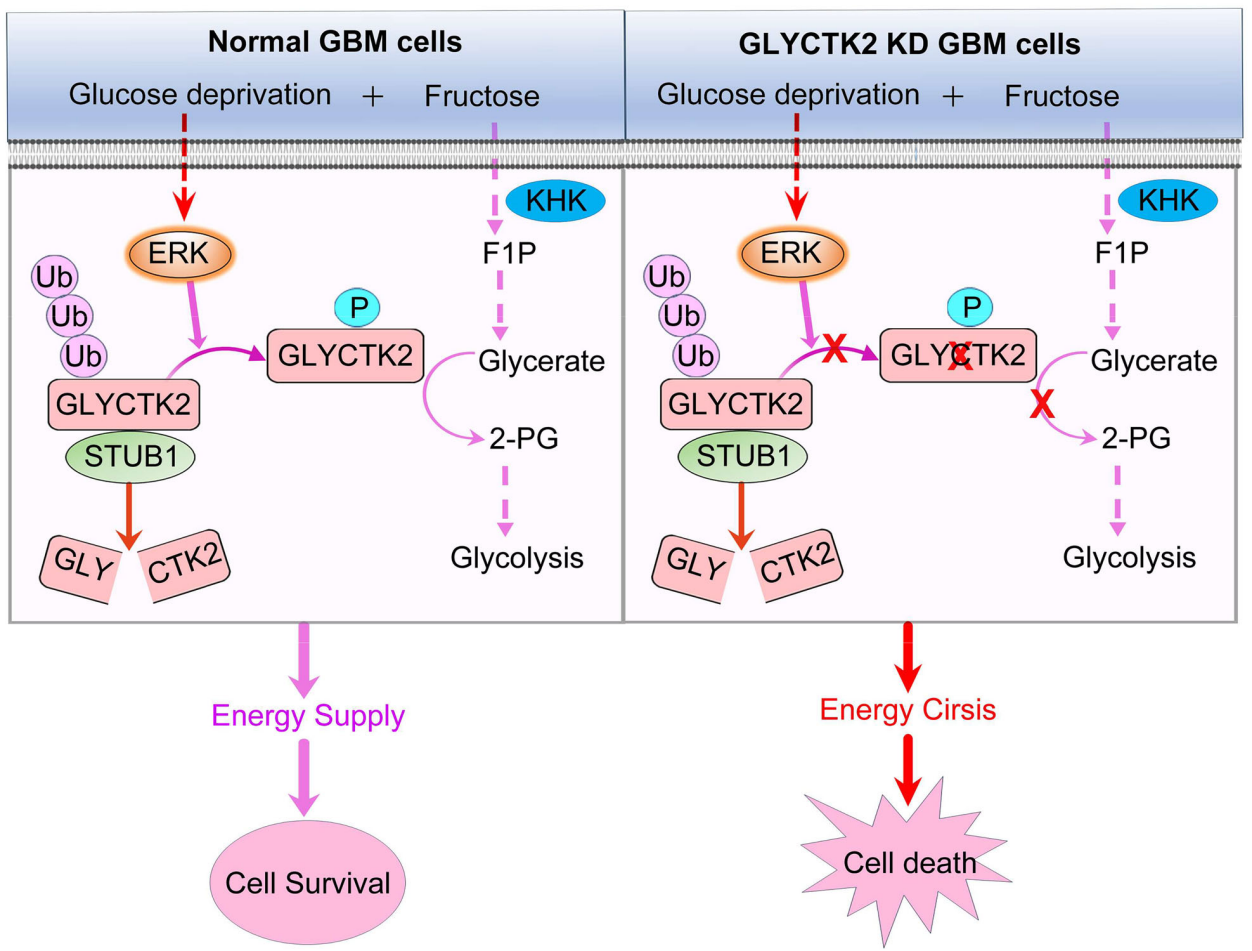
The proposed working model of fructose to maintain GBM cell survival under glucose deprivation via GLYCTK2. Ub ubiquitin, P phosphorylation.

In this context, glycerate kinase 2 (GLYCTK2), a ubiquitously expressed metabolic enzyme responsible for converting glycerol (a fructolysis and serine catabolism byproduct) to 3-phosphoglycerate, warrants investigation. Clinical observations indicate that GLYCTK2 dysfunction causes D-glyceric acidemia, characterized by systemic glycerate accumulation and metabolic acidosis [23–27], highlighting its essential role in intermediary metabolism. Despite these insights, the functional significance of GLYCTK2 in cancer biology, particularly in GBM pathogenesis, remains unexplored. Our study reveals that GLYCTK2 serves as a critical survival mediator for GBM cells under glucose deprivation. Mechanistically, glucose limitation triggers ERK1-mediated phosphorylation of GLYCTK2 at Ser220, which disrupts its interaction with the E3 ubiquitin ligase STUB1, thereby preventing proteasomal degradation and enhancing protein stability. This post-translational stabilization enables GLYCTK2 to sustain energy production through fructose metabolism, promoting tumor cell survival under metabolic stress (Fig. 9). These findings establish GLYCTK2 as a key regulator of fructose utilization in GBM and propose its therapeutic targeting for metabolic intervention.

Notably, while our work identifies Ser220 phosphorylation as a critical determinant of GLYCTK2 stability, several mechanistic questions remain unresolved. First, the structural basis by which phosphorylation modulates the GLYCTK2-STUB1 interaction requires detailed biochemical characterization. Second, the specific lysine residue(s) targeted by STUB1 for ubiquitination remain to be mapped. Addressing these gaps through structural biology approaches and site-directed mutagenesis will refine our understanding of GLYCTK2 regulation and facilitate the development of small-molecule inhibitors. Furthermore, preclinical validation of GLYCTK2 targeting in orthotopic GBM models could strengthen its translational relevance. Collectively, our findings unveil a novel metabolic adaptation axis in GBM and provide a framework for targeting fructose-driven malignancy.

## Materials and methods

### Cell lines and culture conditions

The HEK293T, LN18 and LN229 cell lines were acquired from the American Type Culture Collection (ATCC, USA). U87 and U251 glioblastoma cells were obtained from Cell Bank of Chinese Academy of Sciences (Shanghai, China). The human astrocyte HEB cell line was a gift from Professor Zhimin Lu (Institute of Translational Medicine, Zhejiang University School of Medicine, Hangzhou, Zhejiang, China). All cells used in this study were cultured in Dulbecco’s modified Eagle medium (DMEM; Solarbio, Cat# 12100) containing 25 mM glucose, 4 mM l-glutamine, and sodium pyruvate-free formulation, supplemented with 10% fetal bovine serum (FBS; Excell Bio, Cat# FSP500) at 37 °C with 5% CO_2_. For glucose deprivation studies, confluent cells were first rinsed twice with phosphate-buffered saline (PBS) to remove residual glucose, then incubated in glucose-free DMEM (meilunbio, Cat# MA0587) supplemented with 10% FBS. Experimental groups received 10 mM D-fructose (Sigma-Aldrich, Cat# F3510) supplementation as indicated times. All metabolic interventions were performed under standard culture conditions for specified durations.

### Reagents

Antibodies against GLYCTK (58-964) were purchased from ProSci (Poway, CA, USA). Antibodies against Tubulin (10068-1-AP), Flag (20543-1-AP), Ubiquitin (10201-2-AP), HA (510624-2-AP) and GLUT5 (27571-1-AP) were purchased from Proteintech (Wuhan, China). Anti-KHK rabbit polyclonal antibody (D264350-0025) was purchased from BBI Life Sciences (Shanghai, China). MG132 (S2619), Cycloheximide (S7418), SB202190 (S1077), Compound C (S7840), U0126-EtOH (S1102), H89 (S1582), TCS7010 (S1451), SP600125 (S1460), KU-55933 (S1092) and PF-04691502 (S2743) were purchased from Selleck (Shanghai, China). Fructose (F3510) was purchased from Sigma-Aldrich (Shanghai, China). Propidium iodide (P3566) was purchased from Invitrogen (Shanghai, China). Active GST-ERK1 (#M29-10G) was purchased from SignalChem (Richmond, BC, Canada). ATPγS (ab138911) and p-Nitrobenzyl mesylate (ab138910) were purchased from Abcam (Shanghai, China).

### Plasmids construction

Full-length human *GLYCTK2* (glycerate kinase 2), *STUB1* (STIP1 homology and U-box containing protein 1), and *CBLB* (Casitas B-lineage lymphoma proto-oncogene b) cDNAs were PCR-amplified and cloned into PCMV5-HA, pCDH-SFB/Flag puromycin or pcDNA 3.1-(Flag or HA) vectors. For RNA interference experiments, shRNA oligonucleotides targeting *GLYCTK2* (sense strand: 5′-ATCTCAGGTGGTGAGCCTCAT-3′) were designed using the Broad Institute TRC algorithm and cloned into the pLKO.1-puromycin lentiviral vector.

### Lentivirus package and infection

HEK293T cells were seeded in 100 mm culture dishes and allowed to adhere for 24 h under standard culture conditions (37 °C, 5% CO_2_). Lentiviral production was achieved by co-transfecting 8 μg of either pLKO.1-puro-GLYCTK2 shRNA or pCDH-SFB-GLYCTK2 with packaging plasmids psPAX2 and pMD2.G at a 2:1:1 ratio. Viral supernatants were harvested 48 h post-transfection, centrifuged at 500 × *g* for 5 min to remove cellular debris, and filtered through 0.45 μm filter. Target cells were incubated with viral suspension for 24 h supplemented with 5 μg/mL polybrene in the incubator followed by medium replacement. Selection of transduced cells was initiated 24 h post-infection using 2 μg/mL puromycin for 72 h. Successful knockdown or overexpression was confirmed by western blotting.

### Crystal violet staining

Cells cultured in 6-well plates were gently washed twice with ice-cold phosphate-buffered saline (PBS) to remove residual medium. Fixation was performed by incubating cells with 4% paraformaldehyde (PFA) for 15 min at room temperature. Following fixation, cells were rinsed three times with PBS (5 min per wash) to ensure complete removal of PFA. For morphological staining, fixed cells were incubated with 0.1% (w/v) crystal violet dissolved in 20% methanol for 5 min at room temperature. Excess dye was removed by sequential washing with PBS until the solution became clear. Stained cells were air-dried and photographed. The crystal violet-stained cells were eluted with 10% glacial acetic acid, absorbance of the retained crystal violet was quantified at 595 nm using a microplate reader. Data from three independent experiments were normalized to untreated controls and statistically analyzed using GraphPad Prism 10.

### Cell death analysis

Cultured cells (including dead cells in culture medium) were collected and washed with PBS followed by staining with 10 μg/mL Propidium iodide (PI) in 500 μL PBS for 15 min in the dark. Following staining, cells were pelleted by centrifugation (300 × *g*, 2 min), washed twice with PBS, and finally resuspended in 500 μL PBS. Flow cytometry was performed within 1 h using a CytoFLEX S flow cytometer (Beckman Coulter) equipped with 488 nm excitation and 617 nm emission filters.

### Immunoprecipitation

Cellular proteins were extracted using ice-cold lysis buffer (20 mM Tris-HCl, 150 mM NaCl, 1 mM EDTA, 1 mM EGTA, 2.5 mM sodium pyrophosphate, 1% Triton X-100, pH 7.5) supplemented with 1 mM phenylmethylsulfonyl fluoride (PMSF) as protease inhibitor. For ubiquitination analysis, 20 mM N-ethylmaleimide (NEM) was additionally included to inhibit deubiquitinating enzymes. Following 15-30 min incubation on ice, lysates were transferred to 1.5 mL microcentrifuge tubes and centrifuged at 12,000 × *g* for 15 min at 4 °C. The resulting supernatants were subjected to immunoprecipitation by incubation with protein A/G agarose beads conjugated with specific antibodies under constant rotation at 4 °C for 3 h. Bead-bound immunocomplexes were subsequently washed three times with lysis buffer under stringent conditions (10 min/wash at 4 °C). Immunoprecipitated proteins were eluted by boiling in 2 × SDS loading buffer (62.5 mM Tris-HCl, pH 6.8, 2% SDS, 10% glycerol, 0.01% bromophenol blue) supplemented with 5% β-mercaptoethanol for 10 min at 100 °C. Protein samples were resolved by SDS-PAGE and transferred to PVDF membranes for immunoblotting analysis using designated indicated antibodies.

### Fructose uptake assay

Cells were subjected to glucose deprivation for 6 h, followed by incubation with 10 mM fructose for an additional 6 h. Culture supernatants were collected, and residual fructose levels were quantified using a Fructose Assay Kit (A085-1-1, Nanjing Jiancheng Bioengineering Institute, Nanjing, China) according to the manufacturer’s protocol. Fructose uptake was calculated by subtracting the residual fructose concentration from the initial concentration (10 mM).

### Xenograft mice model

Nude mice model was conducted as described previously [41] with minimal change. Briefly, BALB/c male nude mice (5–6 weeks old, 16–20 g) were obtained from GemPharmatech Co., Ltd (Nanjing, China) and the animal experiment was approved by the Animal Ethics Committee of Nanchang University (acceptance no.: NCULAE-20241010002). Mice were randomly assigned to experimental groups and U87 cells (5 × 10^6^ cells) were harvested and suspended in 100 μL PBS and subcutaneously inoculated into the posterior flanks of nude mice. The mice were maintained in SPF room for 16–18 days and then sacrificed and the tumor pictures and tumor weight were collected. Tumor length (L, longest axis) and width (W, perpendicular axis) were recorded, and tumor volume was calculated using the formula: volume = (length) × (width) ^2^ × 0.5.

### Statical analysis

All quantitative data are presented as the mean ± SEM of at least three independent experiments. The statistical analysis of two-group comparison was analyzed using the Student’s *t* test and the multiple-group comparison was performed using the one-way ANOVA followed by Tukey post hoc test. The average values obtained in the control and experimental groups were analyzed for significant differences. Values of *p* < 0.05 were considered significant.

## Supplementary information

Supplementary figures and figure legends

Unprocessed WB image

## Data Availability

All data and materials of this study are available from the corresponding author upon reasonable request.
