# Supplementary material for: ERK1-mediated GLYCTK2 phosphorylation promotes fructolysis to sustain glioblastoma survival under glucose deprivation
Source: Cell Death Discov. 2025 Jun 4;11:266. doi: 10.1038/s41420-025-02544-3 (PMC12137673; doi:10.1038/s41420-025-02544-3)
Supplement: Supplementary file 1 — Supplementary figures and figure legends [file 41420_2025_2544_MOESM1_ESM.docx]

**Supplementary figures and figure legends for Li et. al.**

**Supplementary Figure Legends**

**Supplementary Figure 1.** **Differential expression of GLYCTK across a panel of tumors** Transcriptomic data from the GEPIA database show elevated *GLYCTK* expression in tumor tissues compared to paired normal counterparts (denoted by red arrowhead).

**Supplementary Figure 2.** **GLYCTK2 governs metabolic adaptation to glucose deprivation in glioblastoma cells**

**A** Time-dependent effects of glucose deprivation on SLC2A5 and KHK expression. Control and GLYCTK2-knockdown U87 cells were exposed to glucose deprivation for 0, 1, 3, or 6 h. Protein levels of SLC2A5 and ketohexokinase (KHK) were analyzed by Western blot, with Tubulin serving as a loading control.

**B** Impaired fructose utilization in GLYCTK2-deficient U87 cells under glucose deprivation. Control and GLYCTK2-knockdown U87 cells were subjected to glucose deprivation for 6 h, followed by supplementation with 10 mM fructose for an additional 6 h. Fructose content in the culture supernatant was quantified to assess cellular fructose consumption. Data are presented as mean ± SEM (n = 3 independent experiments). **p* < 0.05 (Student’s t-test).

**Supplementary Figure 3.** **Ubiquitin E3 ligase CBLB has no effect on GLYCTK2 protein stability**

U87 cells transfected with indicated plasmids were treated with CHX (100 μg /mL) for the indicated time and harvested for immunoblot analysis with indicated antibodies.

**Supplementary Figure 4.** **Predication of GLYCTK2 phosphorylation**

GLYCTK2 phosphorylation was predicted by Scansite 4.0. S220 was predicted as a phosphorylation site of GLYCTK2 by ERK kinase.


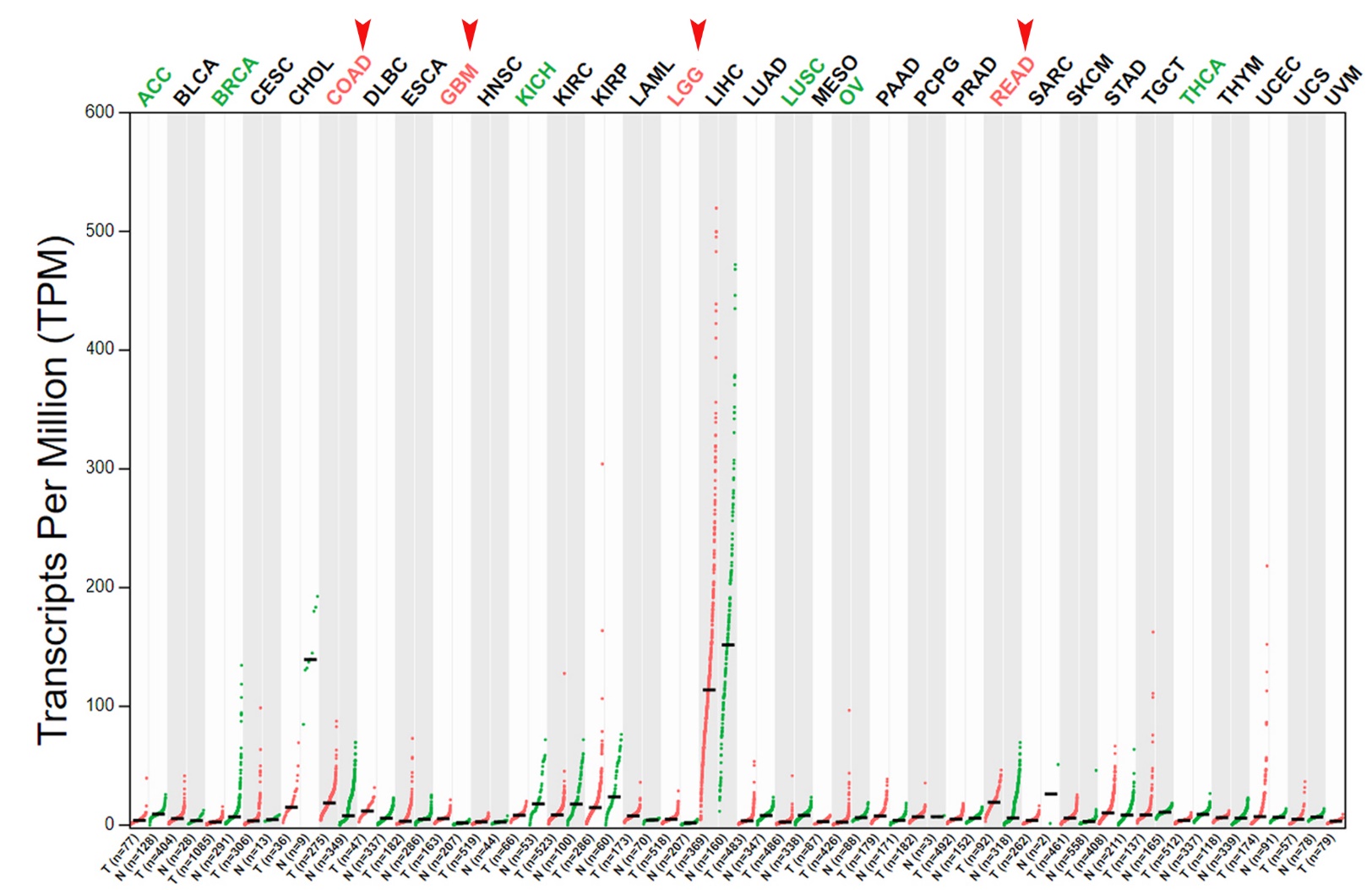


**Supplementary Figure 1**


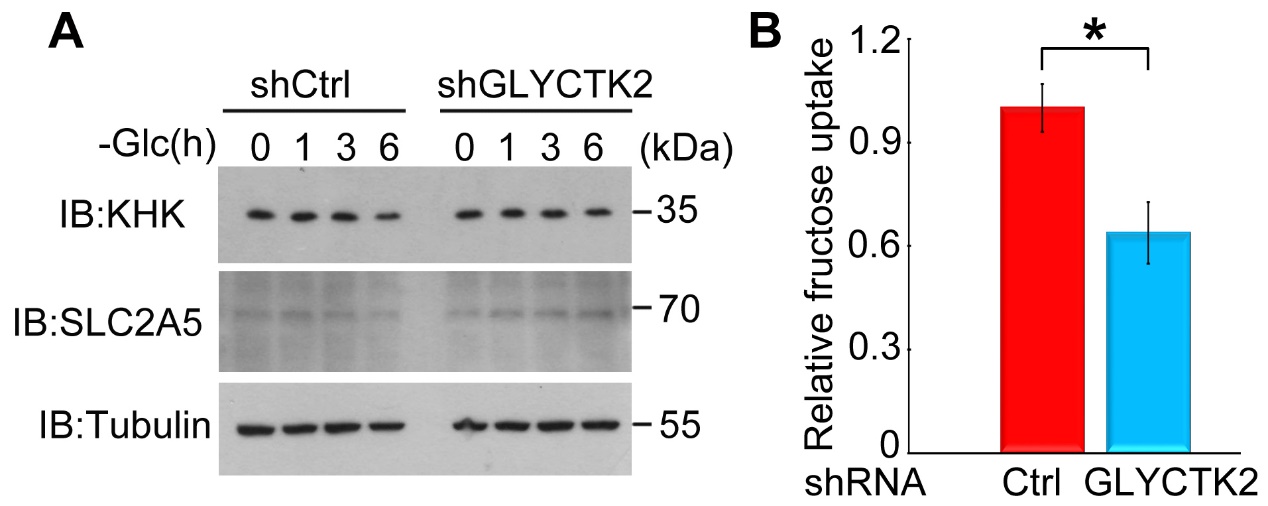


**Supplementary Figure 2**


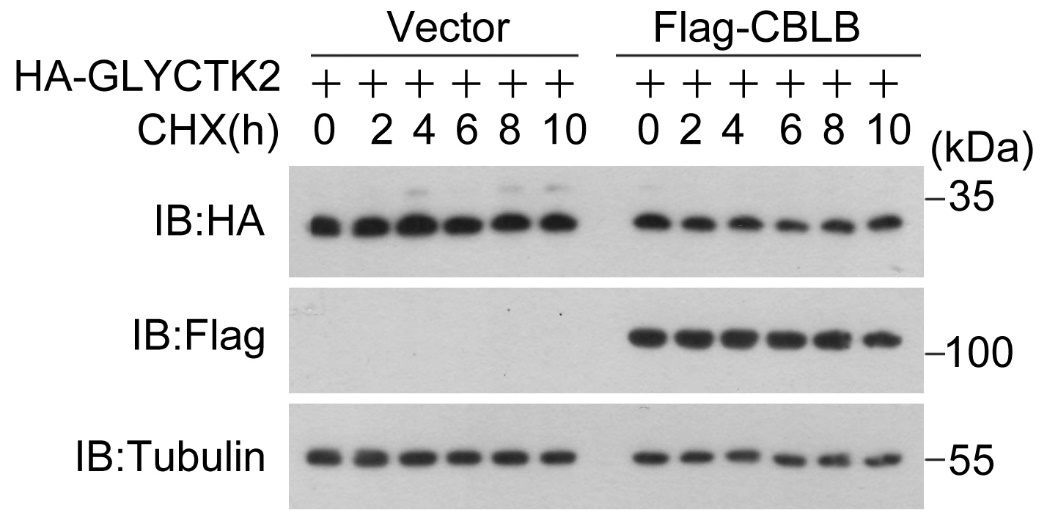

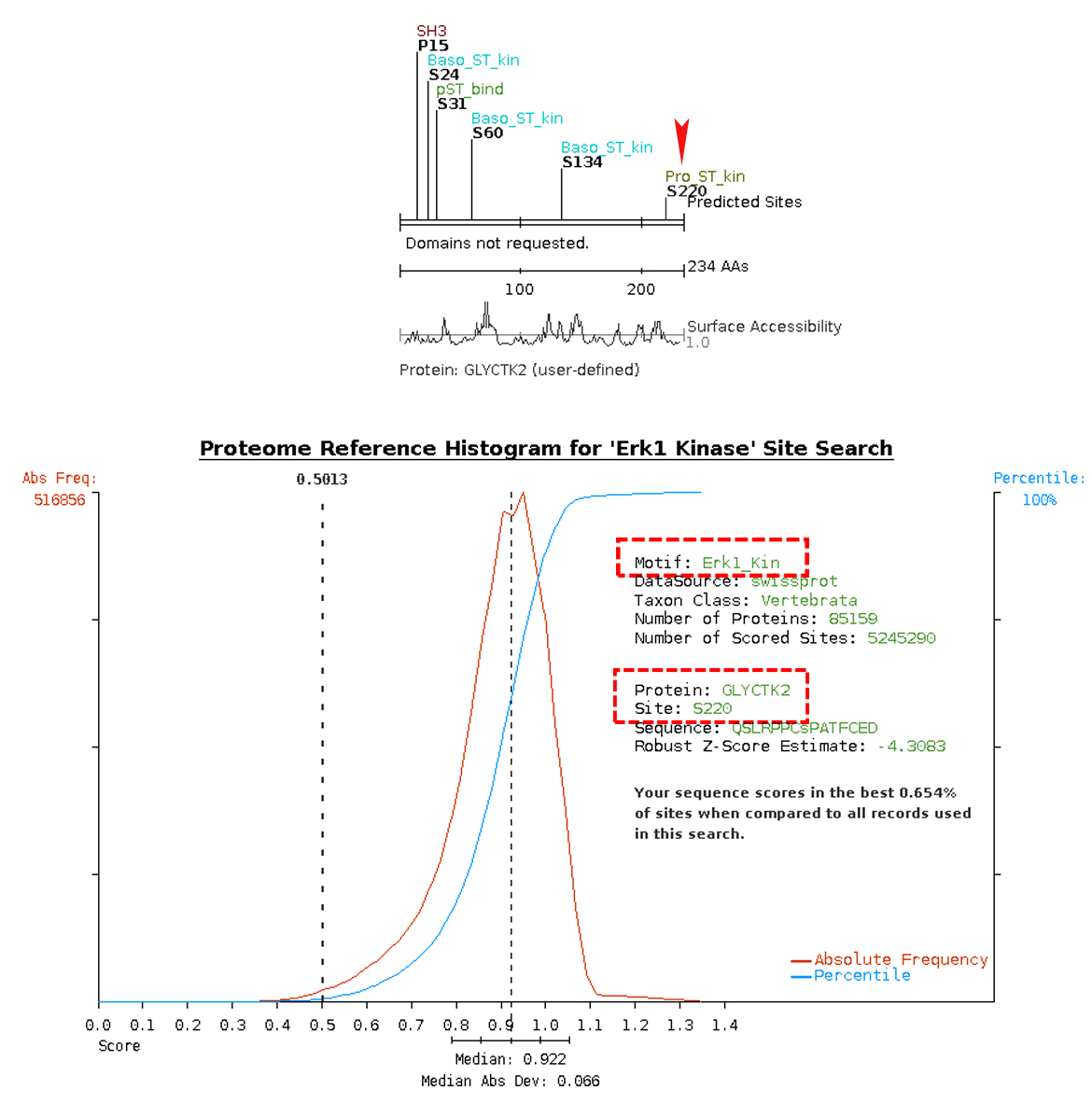
**Supplementary Figure 3**

**Supplementary Figure 4**
