# Supplementary material for: ERK1-mediated GLYCTK2 phosphorylation promotes fructolysis to sustain glioblastoma survival under glucose deprivation
Source: Cell Death Discov. 2025 Jun 4;11:266. doi: 10.1038/s41420-025-02544-3 (PMC12137673; doi:10.1038/s41420-025-02544-3)

Figure 1

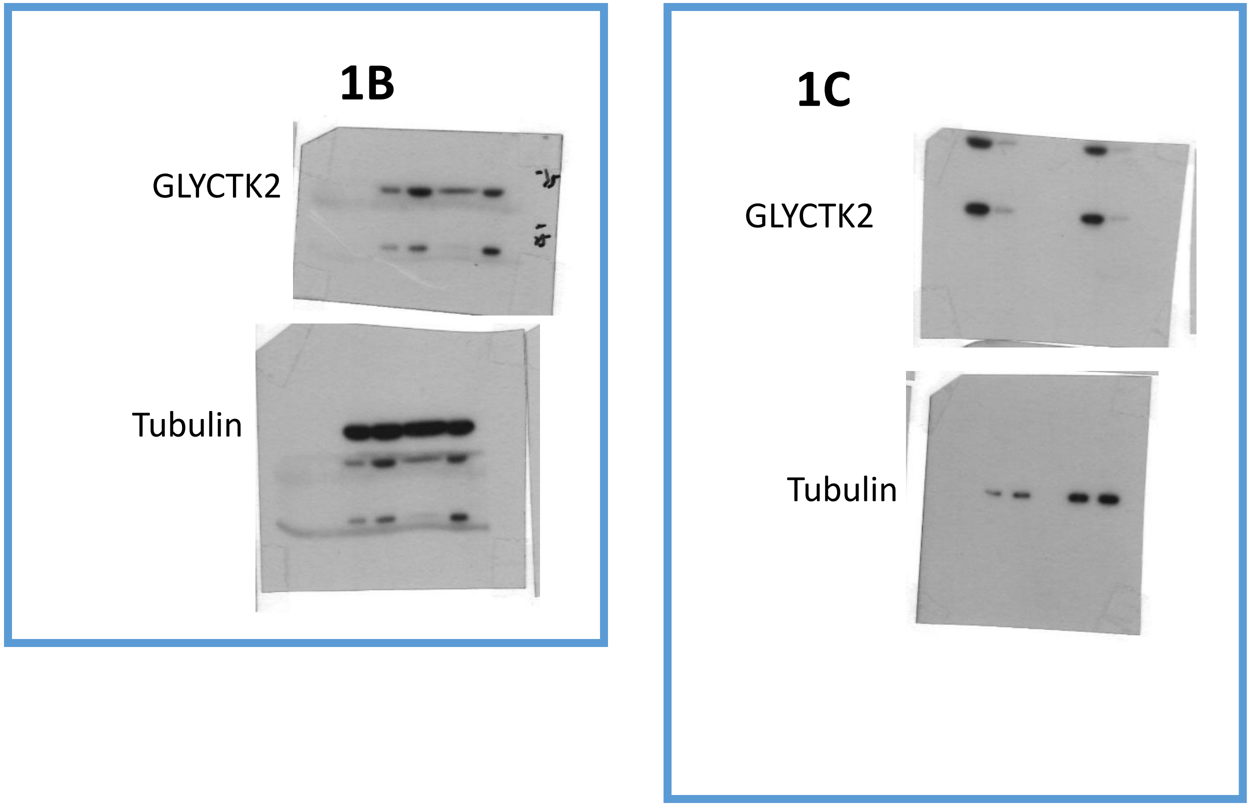

Figure 4

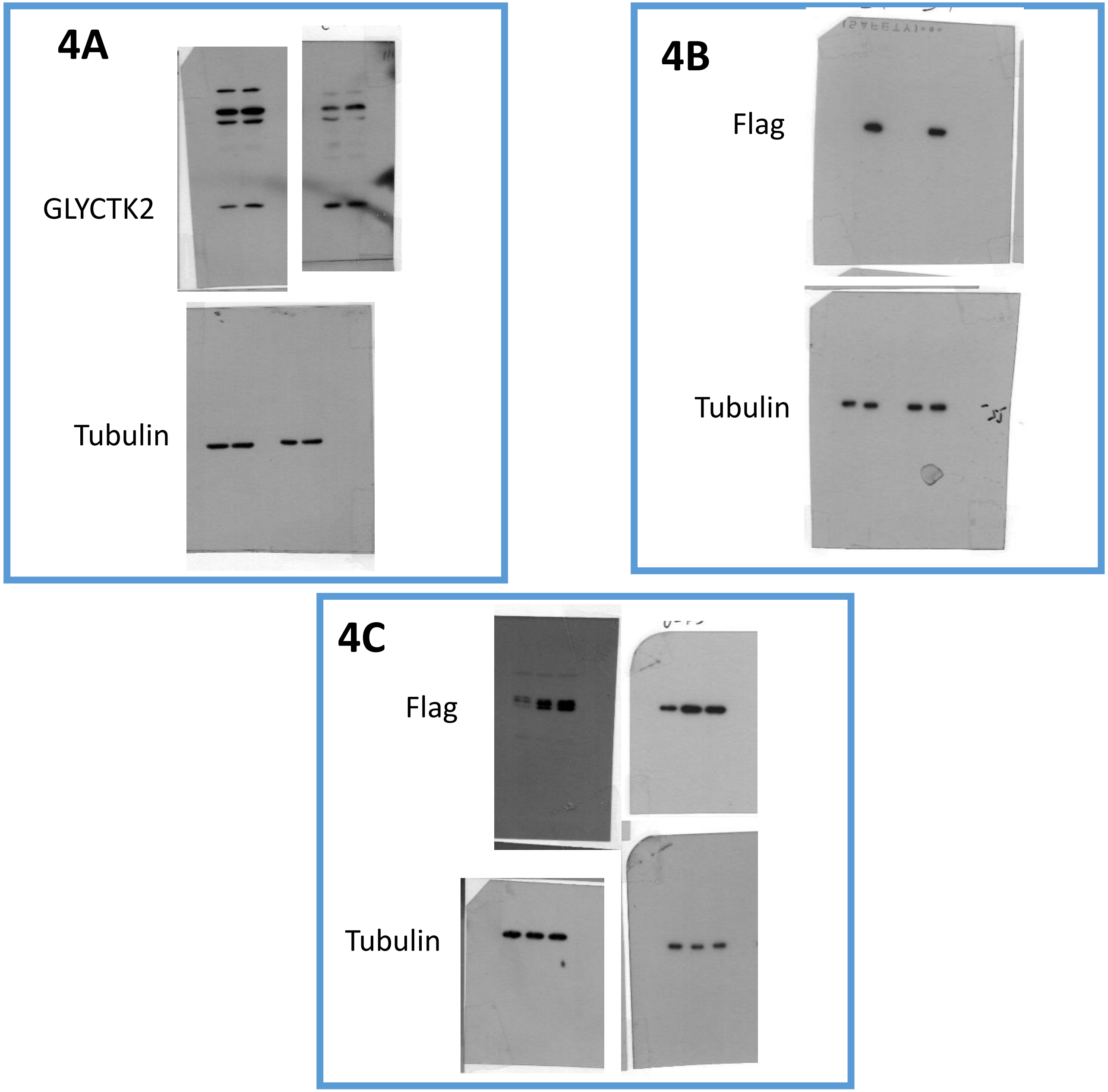

Figure 4

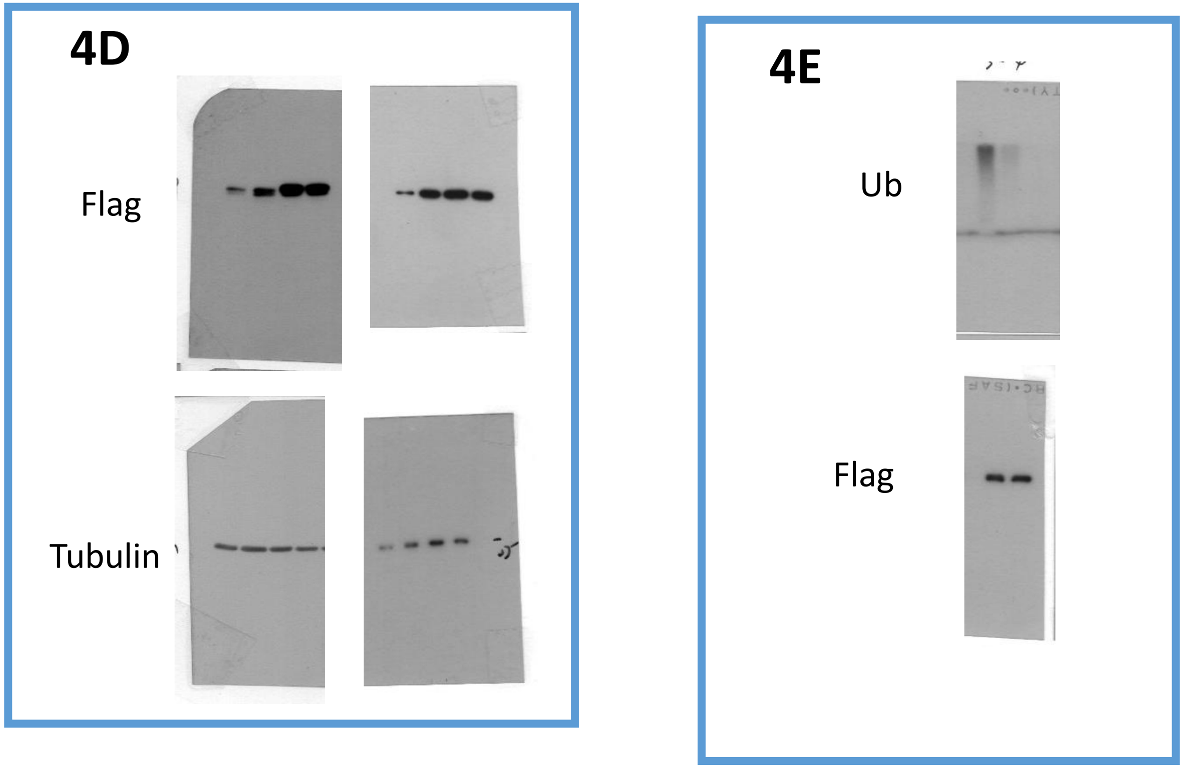

Figure 5

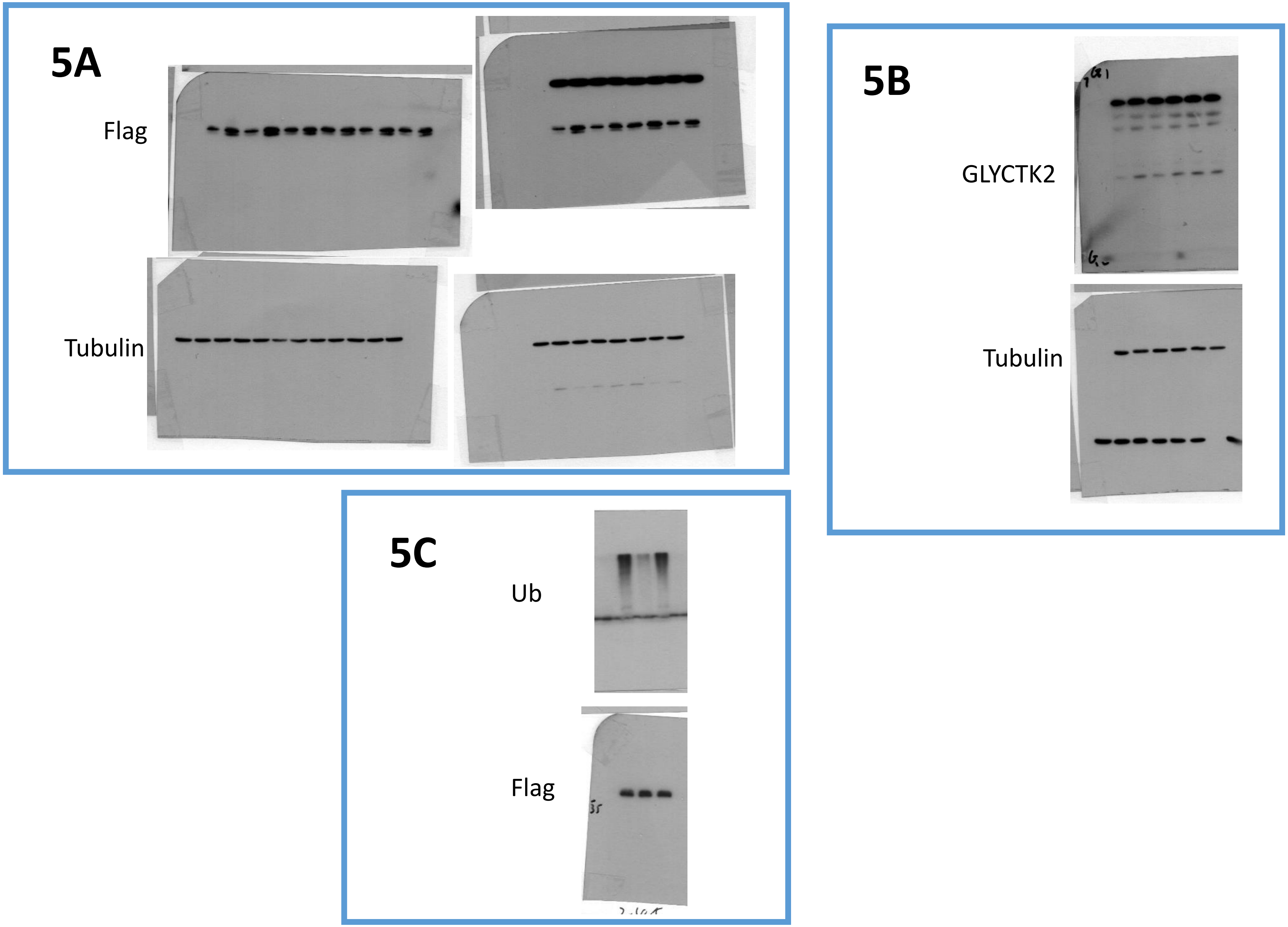

Figure 6

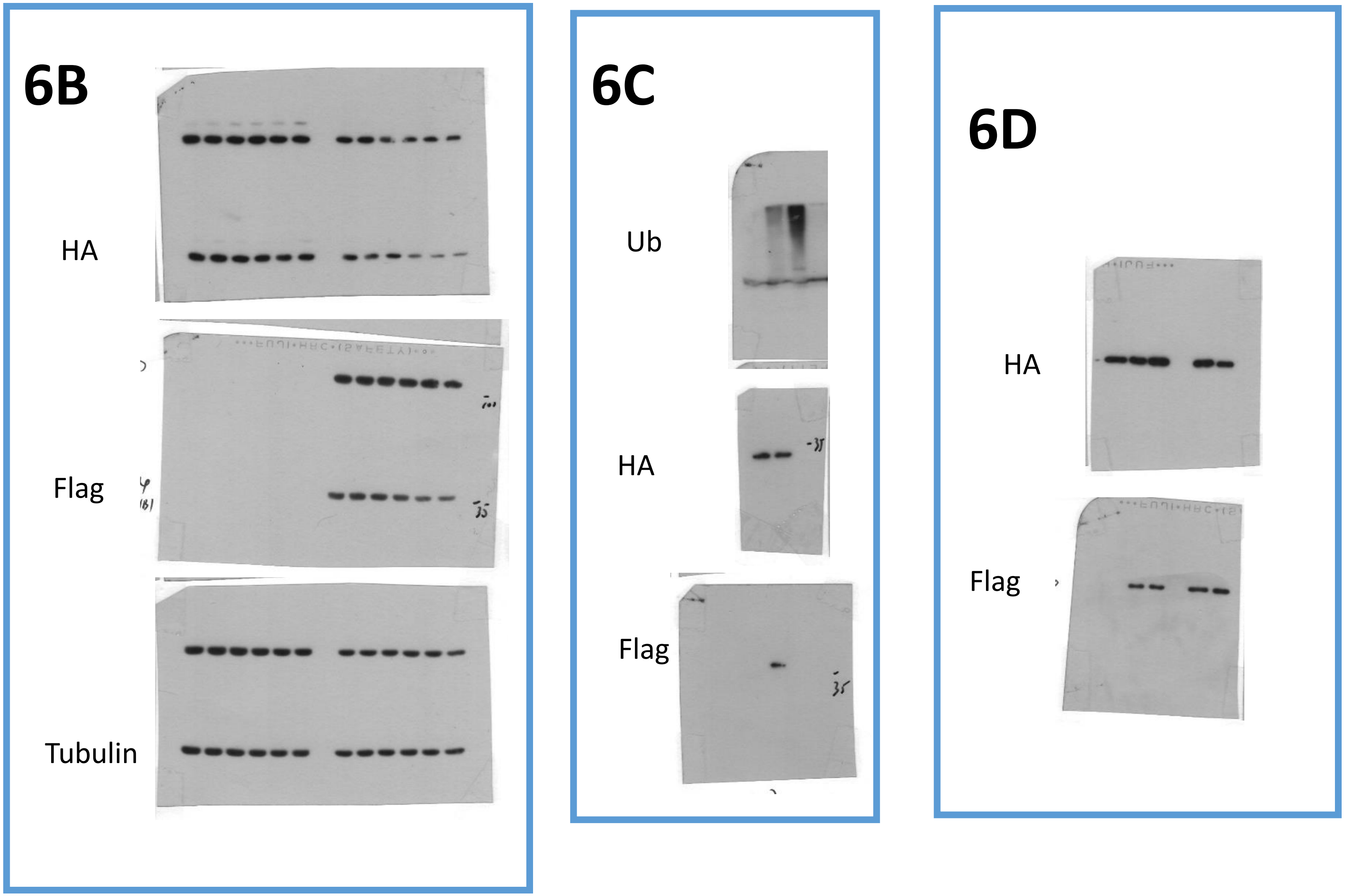

Figure 7

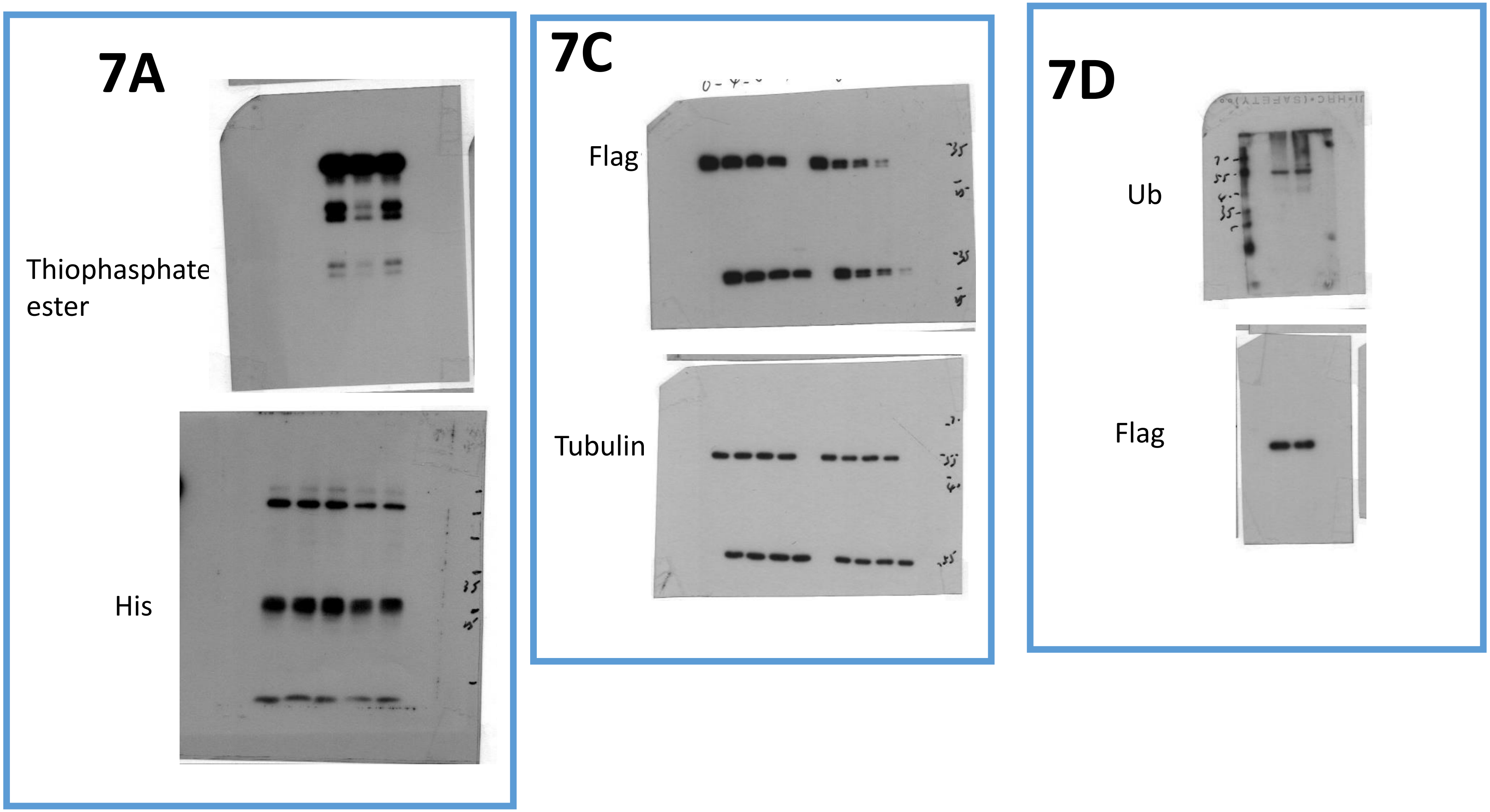

Figure 7 and Figure 8

Figure 7E

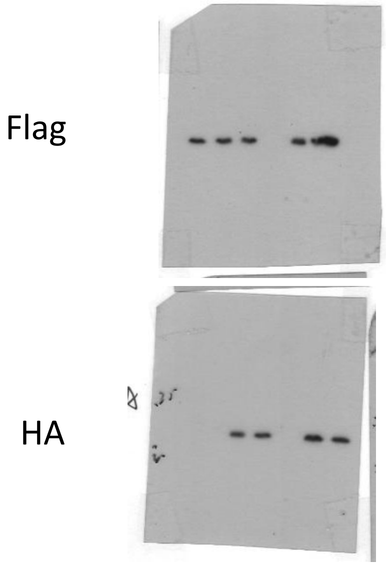

Figure 8A

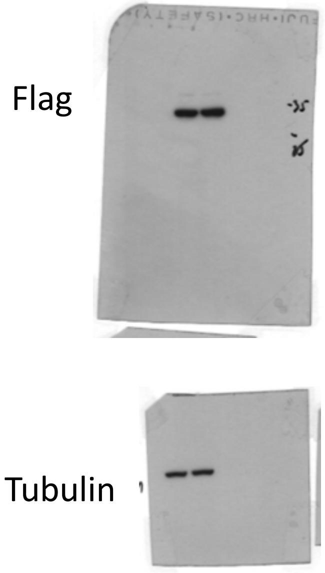

Supplemental Figure 2A

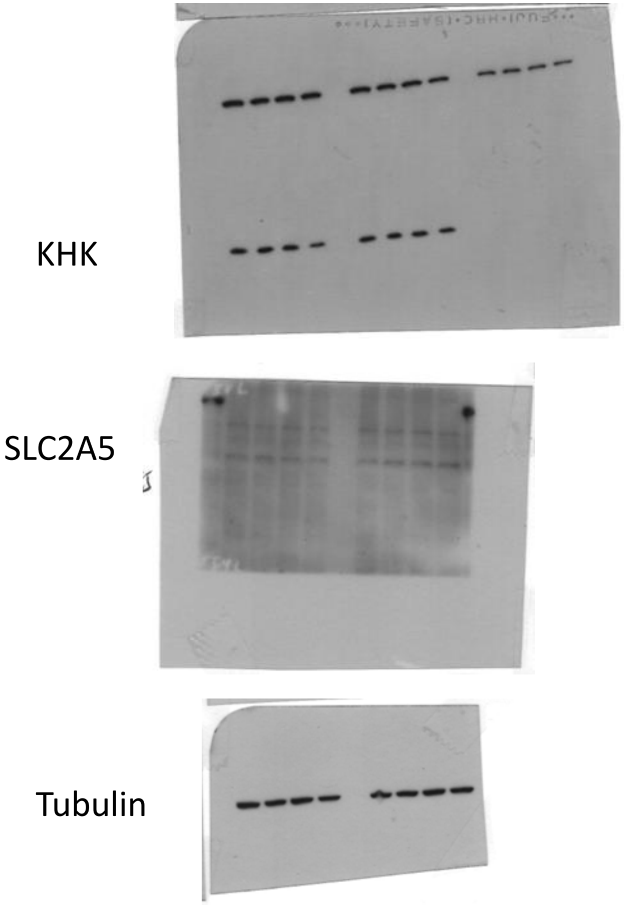

Supplemental Figure 3

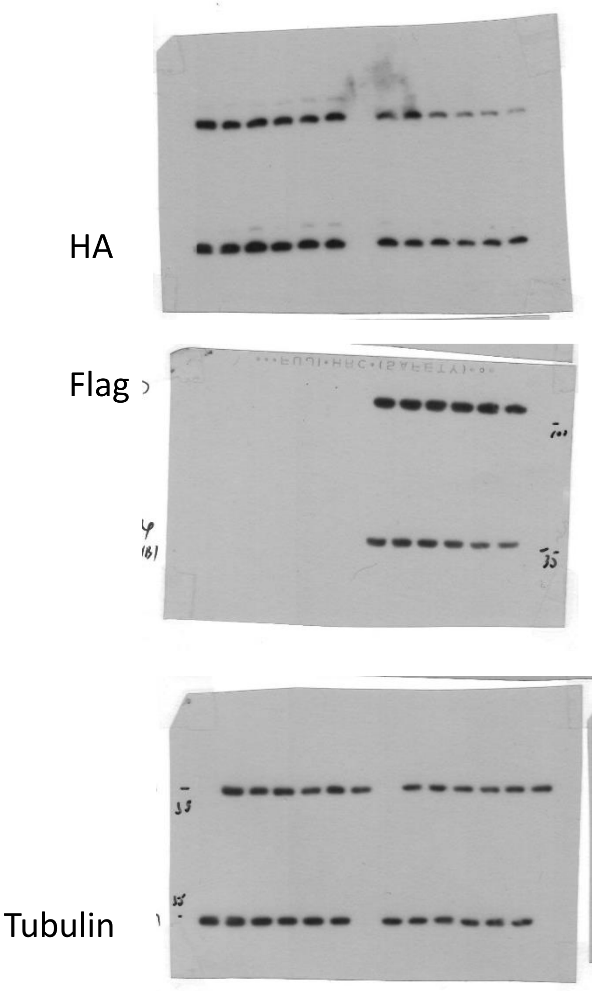

Supplement: Supplementary file 2 — Unprocessed WB image [file 41420_2025_2544_MOESM2_ESM.pdf]
